# Induction of Autophagy Is an Early Response to Gefitinib and a Potential Therapeutic Target in Breast Cancer 

**DOI:** 10.1371/journal.pone.0076503

**Published:** 2013-10-11

**Authors:** Wieslawa H. Dragowska, Sherry A. Weppler, Jun Chih Wang, Ling Yan Wong, Anita I. Kapanen, Jenna S. Rawji, Corinna Warburton, Mohammed A. Qadir, Elizabeth Donohue, Michel Roberge, Sharon M. Gorski, Karen A. Gelmon, Marcel B. Bally

**Affiliations:** 1 Department of Experimental Therapeutics, British Columbia Cancer Agency, Vancouver, British Columbia, Canada; 2 Department of Biochemistry and Molecular Biology, University of British Columbia, Vancouver, British Columbia, Canada; 3 Center for Drug Research and Development, Vancouver, British Columbia, Canada; 4 Michael Smith Genome Sciences Centre, British Columbia Cancer Agency, Vancouver, British Columbia, Canada; 5 Molecular Biology and Biochemistry, Simon Fraser University, Burnaby, British Columbia, Canada; 6 Medical Oncology, British Columbia Cancer Agency, Vancouver, British Columbia, Canada; 7 Department of Medicine, University of British Columbia, Vancouver, British Columbia, Canada; 8 Department of Pathology and Laboratory Medicine, University of British Columbia, Vancouver, British Columbia, Canada; 9 Faculty of Pharmaceutical Sciences, University of British Columbia, Vancouver, British Columbia, Canada; Complutense University, Spain

## Abstract

Gefitinib (Iressa^®^, ZD1839) is a small molecule inhibitor of the epidermal growth factor receptor (EGFR) tyrosine kinase. We report on an early cellular response to gefitinib that involves induction of functional autophagic flux in phenotypically diverse breast cancer cells that were sensitive (BT474 and SKBR3) or insensitive (MCF7-GFPLC3 and JIMT-1) to gefitinib. Our data show that elevation of autophagy in gefitinib-treated breast cancer cells correlated with downregulation of AKT and ERK1/2 signaling early in the course of treatment. Inhibition of autophagosome formation by BECLIN-1 or ATG7 siRNA in combination with gefitinib reduced the abundance of autophagic organelles and sensitized SKBR3 but not MCF7-GFPLC3 cells to cell death. However, inhibition of the late stage of gefitinib-induced autophagy with hydroxychloroquine (HCQ) or bafilomycin A1 significantly increased (p<0.05) cell death in gefitinib-sensitive SKBR3 and BT474 cells, as well as in gefitinib-insensitive JIMT-1 and MCF7-GFPLC3 cells, relative to the effects observed with the respective single agents. Treatment with the combination of gefitinib and HCQ was more effective (p<0.05) in delaying tumor growth than either monotherapy (p>0.05), when compared to vehicle-treated controls. Our results also show that elevated autophagosome content following short-term treatment with gefitinib is a reversible response that ceases upon removal of the drug. In aggregate, these data demonstrate that elevated autophagic flux is an early response to gefitinib and that targeting EGFR and autophagy should be considered when developing new therapeutic strategies for EGFR expressing breast cancers.

## Introduction

Evidence suggests that overexpression and co-expression of EGFR, HER2 and HER3, members of the EGFR receptor family, are associated with resistance to anti-cancer treatments and unfavorable clinical prognosis in breast cancer [1-3]. Therefore, small molecule inhibitors selective for the tyrosine kinases of the EGFR receptor family are of clinical interest [1,2,4,5]. For example, the EGFR tyrosine kinase inhibitor (TKI) gefitinib [6] has been extensively investigated and studies suggested that this drug can be effective against breast cancers expressing EGFR, especially in the background of HER2 overexpression [7-9]. Gefitinib inhibits growth of cancer cells mainly through cytostatic mechanisms, such as G_0_/G_1_ cell cycle arrest and downregulation of cyclin D1 [8], and decreases activation of the phosphatidylinositol 3-kinase (PI3K)/AKT and the mitogen-activated protein kinase (MAPK) pathways [7,8,10]. Gefitinib effects also involve secondary targets, such as protein kinases RICK, GAK and BRK [11]. Here, we report on an additional effect of gefitinib which relates to altering the cellular process of autophagy in breast cancer cells. 

 Macroautophagy (called here autophagy) is an evolutionarily conserved lysosomal degradation pathway executed by the autophagy related (*ATG*) genes [12]. It is a dynamic process starting with the formation of autophagosomes capturing cellular organelles or parts of cytoplasm and leading to fusion with lysosomes, where the autophagosomal cargo undergoes catabolism by hydrolases for recycling of macromolecules [12,13]. This process, called autophagic flow or flux, can be drastically elevated in cells stressed by starvation, hypoxia, radiation, growth factor signaling inhibitors, classical chemotherapy and targeted drugs [14-22]. The role of autophagy in response to anti-cancer therapeutics is, however, not yet well understood. Recent studies suggested that autophagy plays a pro-survival (cytoprotective) role in cancer cells undergoing various anti-cancer treatments [15,17,18,22-26]. This, in turn, may be linked to poor treatment outcomes and development of resistance [17,19,27,28]. It is therefore not surprising that there is a growing interest in autophagy as a putative target for anti-cancer therapy [27-32].

 Using image-based High Content Analysis (HCA), Transmission Electron Microscopy (TEM) and molecular methods we show that gefitinib induces autophagy in various gefitinib-sensitive and -insensitive breast cancer cell lines. Treatment with gefitinib in the presence of lysosomotropic agents that inhibit late-stage autophagy increased efficacy of gefitinib *in vitro* and *in vivo*. Our data support the therapeutic utility of combination treatment strategies based on targeting EGFR and autophagy in breast cancer. 

## Materials and Methods

### Cells and reagents

 SKBR3 and BT474 cells were from the American Type Culture Collection (ATCC), and JIMT-1 cells [33] were purchased from the German Collection of Microorganisms and Cell Culture (Deutsche Sammlung von Mikroorganismen und Zellkulturen GmbH (DSMZ)). SKBR3 cells were maintained in McCoy’s 5A and BT474 and JIMT-1 cells in DMEM. MCF7 cells were stably transfected with the enhanced green fluorescent protein (EGFP)-microtubule-associated protein 1 light chain 3B (MAP1LC3B) construct to generate MCF7-GFPLC3 cells as described previously [34]. MCF7-GFPLC3 cells were maintained in RPMI. Cell cultures were supplemented with 2 mM L-Glutamine and 10% fetal bovine serum (FBS). All cell lines were tested Mycoplasma negative by PCR reaction. Gefitinib was purchased from LC Laboratories and hydroxychloroquine (HCQ) from Acros-Fisher. Bafilomycin A1 and 3-methyladenine (3-MA) were from Sigma-Aldrich and anti-GFP antibody was from Roche. Anti-MAP1LC3B (LC3) antibody was from ABcam or from Cell Signaling Technologies. All other antibodies used in this study were from Cell Signaling Technologies.

### High Content Analysis (HCA)

 Cells were plated in triplicate in flat-bottom 96-well plates (Optilux, Falcon, Becton-Dickinson) in the respective medium, allowed to adhere overnight, and then cells were treated with specified drugs the next day. At the end of drug treatment cells were stained *in situ* with vital dyes: DRAQ5 (Biostatus), Hoechst 33342 (Sigma-Aldrich), ethidium homodimer (ETH) (Life Technologies), monodansylcadaverine (MDC) (Sigma-Aldrich) or lysotracker red (LTR) (Life Technologies) and imaged with IN Cell 1000 Analyzer (GE Healthcare). Ten imaging fields per well were acquired for each fluorescent channel. Images were analyzed with the Investigator image recognition software and Multi Target Analysis (MTA) module. The Investigator software was able to identify cells with ~ 95-99 % accuracy. The number of puncta representing cellular organelles, organelle spacing and total organelle area (TOA) per cell measurements were obtained using the optimized algorithms available in the Investigator software. The average TOA per cell represents a relative measure established and optimized by testing image recognition algorithms on images of cells treated with the vehicle control or indicated therapeutic agents and it does not represent an absolute number. The HCA data were exported and processed using Microsoft Excel.

### Flow cytometry

 Cells were plated in their respective medium containing 10% FBS in T25 flasks or 6 cm diameter culture dishes and allowed to adhere overnight. The next day cells were treated with the indicated agents. After 72 h, supernatant from treated cells (harvested to account for floating dead cells) was transferred to a 14 ml tube and combined with adherent cells harvested with 0.25% Trypsin EDTA. For analysis of cell cycle, including the pre-G_0_/G_1_ fraction, cells were washed twice with PBS and 2x10^6^ cells/sample were fixed in 1.8 ml cold (-20°C) 70% ethanol followed by 1 h incubation on ice and 24 h incubation at -20°C. Cells were then collected by centrifugation and stained in PBS buffer containing 50 µg/ml propidium iodide (PI) (Life Technologies) with 1 mg/ml RNAse A (Sigma-Aldrich) and 0.1% Triton X-100 (Bio-Rad) for 15 min at 37°C followed by 1 h incubation on ice. For Annexin V-based apoptosis analysis, cells were washed twice with Hank’s medium without phenol red and pellets were resuspended in Annexin V buffer containing Annexin V-Alexa Flour® 647 (Annexin V-Alexa647; Life Technologies). Samples were then incubated on ice for 30 min and counterstained with PI at a final concentration of 1 µg/ml. Flow cytometric analysis was performed with the FACSCalibur flow cytometer (Becton-Dickinson) and acquired data were analyzed with the Cellquest software (Becton-Dickinson). The gates used for determining the percentage of different cell populations were set based on the background staining in vehicle-treated cells. The PI-positive and Annexin V-negative cells were considered necrotic, the Annexin V-positive cells (containing both PI-positive late apoptotic and PI-negative early apoptotic cells) were considered apoptotic, and the PI-negative and Annexin V-negative cells were considered viable.

### Western blotting

 Cells were plated in 6 cm culture dishes and after overnight adhesion treated with the indicated drugs. After treatment, cells were lysed in lysis buffer containing 50 mM Tris pH 7.4, 150 mM NaCl, 1% NP-40, 0.25% Na-deoxycholate, 1 mM EDTA, 0.1% SDS, and Mini Protease Inhibitor Cocktail tablets (Roche Diagnostics). After centrifugation (30 min at 13,000 rpm) the protein concentration in the supernatant was quantified using the Pierce Micro BCA^TM^ Assay Kit. 30 - 50 µg of total protein per sample was separated on precast 4 - 12% Bis-Tris gels (NuPage, Life Technologies) and transferred to NuPage 0.45 µm nitrocellulose membranes. Membranes were blocked with 5% skim milk powder in 150 mM NaCl with 50 mM Tris and 0.1% Tween-20 at pH 7.4 (TBS-T) and incubated overnight with primary antibodies in 5% BSA in TBS-T. The next day membranes were washed 3 times with TBS-T and incubated for 1 h with peroxidase-conjugated secondary antibodies (Promega) in TBS-T containing 5% skim milk. Membranes were then washed 3 times with TBS-T and signals were detected by enhanced chemiluminescence (SuperSignal® West Pico Chemiluminescent Substrate, Thermo Scientific) on CL-XPosure^TM^ Film (Thermo Scientific) or by acquisition with the ChemiDoc^TM^ MP imaging system (Bio-Rad).

### Small interfering RNA (siRNA)

 Expression of endogenous BECLIN-1 (BECN1), ATG7 and EGFR messages was silenced with chemically modified Stealth™ siRNA (BECN1: 5′-GGAUGAUGAGCUGAAGAGUGUUGAA-3′, ATG7: 5′-CCAAGGAUGGUGAACCUCAGUGAAU-3′, EGFR: 5′-CCUAUGCCUUAG CAGUCUUAUCUAA-3′; Life Technologies/Invitrogen). A scrambled Stealth™ siRNA with medium GC content was used as a negative control (Life Technologies/Invitrogen catalogue 12935-200). To account for any unanticipated off-target effects, each siRNA duplex for the scrambled and targeted genes was tested for induction of an interferon-mediated stress response using methods described previously [15]. Stealth™ siRNA duplexes were delivered to the target cell populations by electroporation using the AMAXA™ Nucleofector™ system (Lonza). SKBR3 cells were electroporated in nucleofector solution C using program E009. MCF7-GFPLC3 cells were electroporated in nucleofector solution V using program P020. In specified experiments, a single siRNA transfection or two serial siRNA transfections 72 h apart (referred to as a “double knockdown”) were performed to achieve knockdown of gene expression. At the indicated time following transfection, cells were plated in 96-well plates for imaging experiments with IN Cell 1000 or in 6 cm culture dishes for Western blot analysis. 

### Quantitative Real-Time PCR (qRT-PCR)

 mRNA expression of BECN1 and ATG7 was assessed by qRT-PCR 48 - 96 h following the final transfection with siRNA. Total RNA was isolated using an RNeasy® Mini kit (Qiagen) and reverse transcribed into cDNA using a QuantiTect Reverse Transcription kit (Qiagen) according to the manufacturer’s instructions. TaqMan® Gene Expression assays (Applied Biosystems) were used to amplify BECN1 (assay ID Hs00186838) and ATG7 (assay ID Hs00197348) cDNA in triplicate single-plex reactions. GAPDH (assay ID Hs02758991) was used as an endogenous reference gene for normalization and relative gene expression was calculated using the standard curve method.

### Transmission Electron Microscopy (TEM)

 Cells were harvested with trypsin EDTA and fixed in 4% formaldehyde and 2.5% glutaraldehyde (GA) in 0.1 M sodium cacodylate buffer (pH 7.4), post fixed in buffered 1% osmium tetroxide, embedded and fixed a second time in 2.5% GA, cut into 1 mm cubes, then dehydrated through a graded ethanol series and propylene oxide prior to microwave infiltration of 1:1 Spurr/Epon resin. Polymerized blocks were sectioned on a Reichert Ultracut E and the 70 nm sections were mounted on 100 mesh grids or 1x2 slot grids, and stained (for 12 or 6 seconds, respectively) in uranyl acetate and Reynolds lead citrate. Images were acquired with a Hitachi H7600 TEM (Tokyo, Japan).

### Clonogenic assay

 500 trypan blue excluding cells were plated in quadruplicates (from two independent dilutions) in 3 ml growth medium in 6-well plates and cultured for 17 days. After removal of medium from the wells, colonies were stained with 0.2% malachite green and counted. Plating efficiency was defined as the percentage of trypan blue excluding cells that formed colonies of >50 cells.

### Tumor xenografts and treatment

 For *in vivo* studies JIMT-1 cells were harvested in the exponential growth phase. 7.5 × 10^6^ JIMT-1 cells were injected subcutaneously (s.c.) on the back of female Rag2M immuno-compromised mice. Tumor growth was monitored twice a week; tumor sizes were calculated using the formula 0.5 [length (mm)] × [width (mm)^2^]. When tumors reached a volume of approximately 100 mm^3^, animals were randomized to different treatment groups (6 animals per group). Treatment was initiated on day 25 and carried out Monday through Friday (QDx5) for 25 or 26 days. All agents were delivered as oral gavage. Gefitinib was solubilized in 0.5% Tween-80 in sterile milli-Q water and HCQ was solubilized in milli-Q water. Gefitinib and HCQ formulations were prepared weekly and kept at 4°C. Combination treated mice were dosed first with gefitinib followed by HCQ four hours later. Animals were also monitored for body weight loss and other signs of sickness due to treatment-related side effects or tumor burden. Animal protocols were approved by the University of British Columbia Animal Care Committee, and these studies were done in accordance with guidelines established by The Canadian Council on Animal Care.

### Statistical analysis

 Differences among the treatment groups were assessed with an unpaired t-test (GraphPad Prism Version 5.00). The obtained p values were adjusted for multiple comparisons using the Benjamini-Hochberg procedure (R version 2.11.1), when applicable. Statistical analysis of differences in tumor volume between different treatment groups was performed using Kruskal-Wallis test with Dunn’s correction for multiple comparisons (GraphPad Prism Version 6.01). Differences were considered significant at p 0.05.

## Results

### Gefitinib treatment induced the appearance of MDC-labeled vesicles in breast cancer cells regardless of their sensitivity to gefitinib

**Figure 1:**
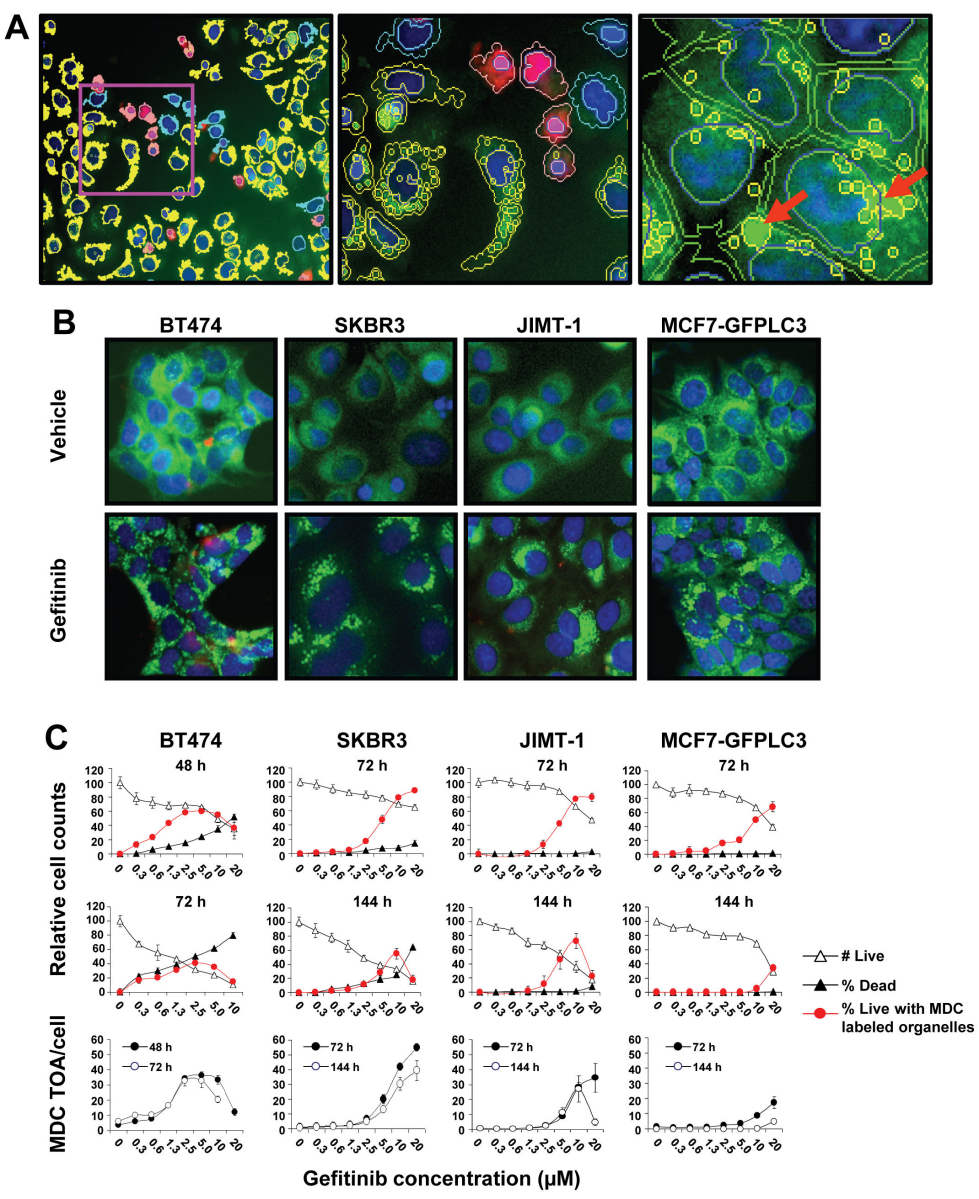
Gefitinib inhibits cell growth and induces appearance of MDC-labeled organelles in breast cancer cells. (**A**) Example images obtained with IN Cell 1000 showing SKBR3 cells treated for 72 h with 10 µM gefitinib and stained *in* *situ* with DRAQ5 (stains nuclei in viable and dead cells), ETH (stains nuclei in dead cells with compromised plasma membrane) and MDC (stains acidic organelles). Based on differential staining and morphological features image recognition software identifies different cell populations. Left image: nuclear imaging masks shown in blue and red indicate viable and dead cells, respectively; cytoplasm of viable cells with MDC-labeled organelles is shown in yellow and cytoplasm of cells without MDC-labeled organelles is shown in light blue. Middle image: magnification of an area, as marked in the left image, showing transparent imaging masks outlining nuclei, cytoplasm and MDC-labeled organelles, as recognized by the HCA Investigator software; DRAQ5 staining is shown in blue, ETH staining is shown in red and MDC staining is shown in green. Right image: a high magnification image showing MDC-labeled organelles outlined in yellow within the cells’ cytoplasm; some of the MDC-labeled structures, indicated by the red arrows, represent multiple closely grouped organelles. (**B**) Representative images of indicated cells cultured in the presence of 0.5% DMSO (vehicle) or 10 µM gefitinib for 24 h (BT474) or 72 h (SKBR3, JIMT-1, MCF7-GFPLC3) stained *in* *situ* with DRAQ5 (blue), ETH (red) and MDC (green). Images in (**A**) and (**B**) were pseudo-colored and overlaid using the Investigator software. (**C**) Quantitation of different cell populations and autophagic organelles in BT474, SKBR3, JIMT-1 and MCF7-GFPLC3 cells by HCA. Cells were treated with vehicle or increasing concentrations of gefitinib for the indicated time. Numbers of viable cells in culture are normalized to vehicle-treated controls. Dead cells are shown as a percent difference in the content of dead (ETH-positive) cells between drug-treated minus vehicle-treated cultures. The proportion of viable cells with MDC-labeled organelles (puncta) in culture is shown as a percent difference in cells with >1 MDC-labeled organelle between drug-treated minus vehicle-treated cultures. Each data point represents a mean±SD from 3 replicate wells. HCA screenings were repeated 2 - 3 times for each cell type with consistent results; representative experiments are shown.

 While implementing an imaging based high content analysis (HCA) screen to look for compounds that interacted synergistically when combined with gefitinib, we noted that gefitinib treatment induced the appearance of vesicular organelles in the cytoplasm of breast cancer cells. When stained with the monodansylcadaverine (MDC) dye these organelles accumulated the stain providing an indication that alterations in autophagy may be occurring [35]. Figure (Fig.) 1A shows images of gefitinib-treated SKBR3 cells with overlaid software-derived imaging masks. These images reveal the heterogeneity of cell populations based on differential labeling of viable and dead cells by DRAQ5 and ETH with MDC-labeled cytoplasmic organelles (green puncta) and show details of cell morphology (left and middle panels). The right panel in Figure 1A illustrates that when individual organelles are positioned in close proximity within cells it is more difficult for the analysis software to recognize individual structures, therefore, the total area occupied by all the labeled organelles within a cell (Total Organelle Area (TOA)) was used as a practical way to quantitate these structures. Following treatment with a range of gefitinib concentrations over time, different cell populations were quantified in phenotypically diverse breast cancer cell lines. SKBR3 cells express a wild-type PIK3CA gene encoding the p110-alpha catalytic subunit of PI3K, and BT474, JIMT-1 and MCF7 cells express a mutated PIK3CA gene. These cells also differ in estrogen receptor (ER), EGFR, HER2 and HER3 expression: SKBR3 cells are ER-negative (ER^-^) and express high levels of HER2 and EGFR and medium levels of HER3, BT474 cells are ER-positive (ER^+^) and express high levels of HER2 and HER3 but low levels of EGFR, JIMT-1 cells are ER^-^ and express high levels of HER2 and EGFR and low levels of HER3, and MCF7 cells are ER^+^ and express low levels of HER2 and EGFR and high levels of HER3 [7,33,36-38]. MCF7 cells used in our study were stably transfected with an ectopic EGFP- LC3B construct to generate MCF7-GFPLC3 cells [34]. Representative images of different cells treated with vehicle or gefitinib and stained *in situ* with DRAQ5, ETH and MDC are provided in Figure 1B. These images show accumulation of MDC-labeled organelles in gefitinib-treated cells. Imaging data were quantified with the Investigator software and the HCA data obtained for the different cell lines are presented in Figure 1C. These data show that gefitinib treatment produced cell populations with MDC-labeled organelles in a concentration-dependent fashion. In BT474 cells, 48 h treatment with gefitinib resulted in cytotoxicity (decrease in viable cells and associated rise in dead cells) occurring at therapeutically relevant drug concentrations (~ 1 µM; [6,39]) which further increased over a period of 72 h (Figure 1C, BT474 top and middle graphs). In SKBR3 cells, cytotoxicity was only apparent after 144 h and when gefitinib was used at concentrations ≥ 1 µM (Figure 1C, SKBR3 top and middle graphs). In JIMT-1 and MCF7GFPLC3 cells, gefitinib-mediated cytotoxicity was negligible even after 144 h and growth inhibition was a result of cytostatic effects (Figure 1C, JIMT-1 and MCF7-GFPLC3 top and middle graphs). Based on the cytotoxicity data, we considered BT474 very sensitive, SKBR3 moderately sensitive and JIMT-1 and MCF7GFPLC3 cells insensitive to gefitinib. The decline in viable cell numbers in all cell lines treated with gefitinib coincided with an increase in cell populations containing MDC-labeled organelles that were associated with cytotoxic and/or cytostatic effects (Figure 1C, top and middle panels). The data presented in Figure 1C in the bottom panel show the average MDC TOA/cell in cells treated with gefitinib. These data correlate well with the proportion of cells with MDC-labeled organelles shown in Figure 1C top and middle panels suggesting that both measures are suitable for the quantitative assessment of cellular organelle content. Together these results show that MDC-labeled organelles accumulate following gefitinib treatment in breast cancer cells with different sensitivities to this drug**.**

### TEM confirms the presence of autophagy-associated organelles in gefitinib-treated cells

**Figure 2:**
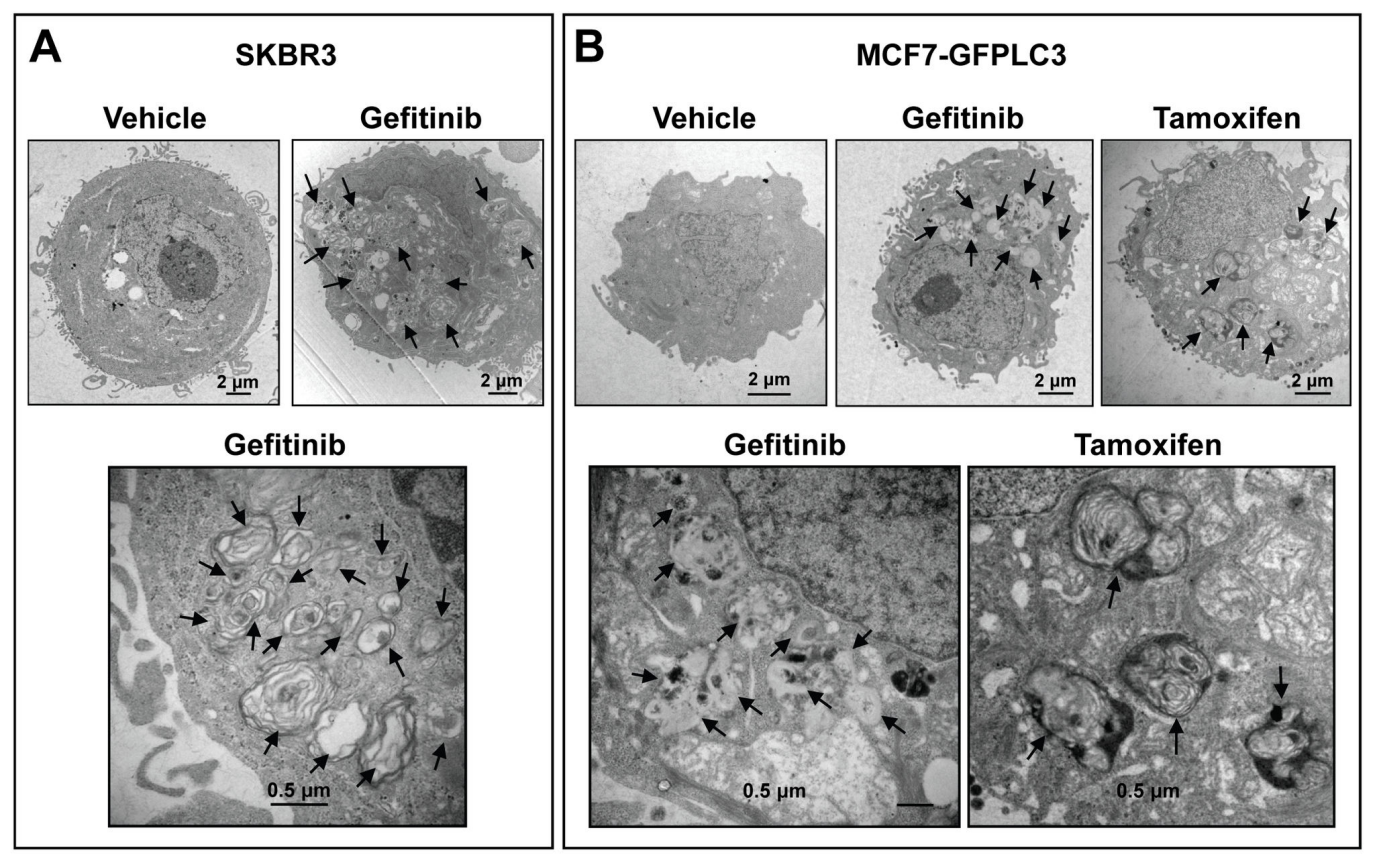
TEM images of breast cancer cells treated with gefitinib or tamoxifen. (**A**) SKBR3 cells treated for 48 h with 0.5% DMSO (vehicle) or 20 μM gefitinib. (**B**) MCF7-GFPLC3 cells treated for 48 h with vehicle, 20 μM gefitinib or 10 μM tamoxifen. High magnification images in (**A**) and (**B**) show details of the cytoplasmic organelles containing membranous and degraded cellular material in double or single membrane bound vesicles indicated by arrows. Representative images are shown.

Since the MDC dye is known to stain acidic organelles such as lysosomes, autolysosomes and late autophagosomes [35], the increase in MDC-labeled vesicles in gefitinib-treated cells suggested that this drug modulates autophagy. To determine whether this is the case, we used TEM, a commonly accepted method for identification of autophagic organelles [35]. These results are summarized in Figure 2 which shows representative TEM images of 48 h vehicle or gefitinib-treated SKBR3 (Figure 2A; an example of a gefitinib-sensitive cell line) and MCF7-GFPLC3 cells (Figure 2B, top left and centre and bottom left images; an example of a gefitinib-insensitive cell line). As a positive control, the ER^+^ MCF7-GFPLC3 cells were treated with tamoxifen (Figure 2B, top and bottom right images), a drug that is known to induce autophagy [15]. The cytoplasm of SKBR3 and MCF7-GFPLC3 vehicle-treated cells was uniform in contrast to the cytoplasm in gefitinib-treated cells which contained numerous autophagosomes and autolysosomes with enclosed degenerative cellular material. The vesicular structures in gefitinib-treated MCF7-GFPLC3 cells were, in general, similar in appearance to those observed in cells treated with tamoxifen. The presence of these structures in SKBR3 and MCF7-GFPLC3 cells following gefitinib treatment correlated well with the existence of MDC or lysotracker red (LTR)-labeled organelles and with the GFPLC3-, LTR- or MDC-labeled organelles, respectively, as shown in Figure S1. 

### Increase in autophagosomes is an early response to gefitinib treatment associated with cell-type specific changes in EGFR signaling

**Figure 3:**
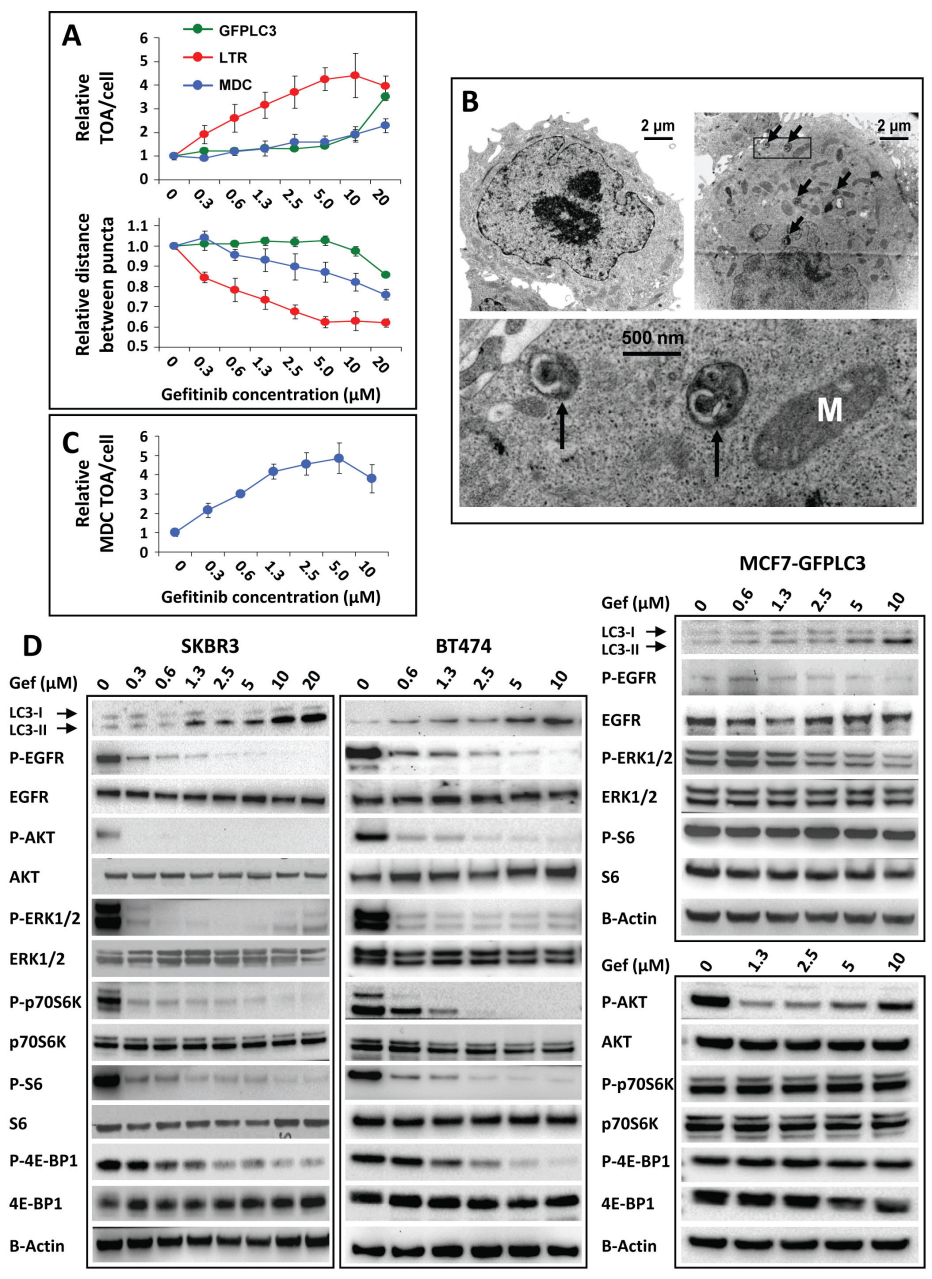
Early responses of breast cancer cells to gefitinib. (**A**) MCF7-GFPLC3 cells treated for 3 h with vehicle (0 µM gefitinib) or indicated doses of gefitinib. Average GFPLC3, lysotracker red (LTR) and MDC TOA per cell (top graph) and the average distance between GFPLC3, LTR or MDC-labeled organelles (puncta) (bottom graph) were normalized to the respective measurements in vehicle-treated cells expressed as 1. (**B**) Representative TEM images of MCF7-GFPLC3 cells treated for 3 h with vehicle (top left image) or 10 µM gefitinib (top right image). A higher resolution image of the marked area in gefitinib-treated cells is shown on the bottom. Arrows indicate electron dense organelles engulfing double membrane vesicles, representing autolysosomes. M: mitochondrion. (**C**) SKBR3 cells treated with indicated doses of gefitinib for 3 h. The average MDC TOA/cell is normalized to vehicle-treated cells expressed as 1. Each data point in (**A**) and (**C**) represents a mean±SD from 3 replicate wells. These experiments were repeated 3 times with consistent results. Representative experiments are shown. (**D**) Western blot analysis of lysates derived from SKBR3 and BT474 cells treated for 3 h and from MCF7-GFPLC3 cells treated for 4 h with increasing concentrations of gefitinib (Gef). B-Actin was used as loading control.

The dynamics of autophagosome formation as a response to gefitinib treatment were investigated in MCF7-GFPLC3 and SKBR3 cells. The GFPLC3 fusion protein, commonly utilized as an autophagosomal marker [35], is typically observed throughout the cytoplasm as diffuse labeling in cells growing under normal conditions. However, conditions that trigger autophagy cause this protein to relocate to the membrane of newly formed autophagosomes which will then appear as green fluorescent puncta representing GFPLC3-labeled organelles. The GFPLC3-labeled organelles in MCF7-GFPLC3 cells started to be visible ~ 45 min after gefitinib treatment was initiated (Figure S2, GFPLC3 top panel). The number of GFPLC3-labeled organelles increased steadily over three hours, as diffuse cytosolic GFPLC3 re-localized to punctate structures (Figure S2, GFPLC3 lower panel). These data strongly suggest that autophagosomes accumulate in gefitinib-treated cells. MCF7-GFPLC3 cells stained with LTR or MDC after 3 h treatment with gefitinib (Figure S2, MDC and LTR panels) showed similar results with the appearance of LTR and MDC-labeled organelles correlating with GFPLC3-labeled autophagosomes. The HCA data indicated that the average total area of these organelles per cell increased in a concentration-dependent manner (Figure 3A, top graph). The image analysis methods used here could also estimate the distance between identified organelles (puncta); these data suggest that the distance between individual organelles decreases in gefitinib-treated cells as the total organelle area increases (Figure 3A, lower graph). It can be suggested that this is a reflection of organelle clustering, a feature that also appears to be concentration-dependent. TEM images of MCF7-GFPLC3 cells treated for 3 h with vehicle show numerous well preserved mitochondria and some immature small lysosomes (Figure 3B, top left image). After 3 h treatment with 10 μM gefitinib, MCF7-GFPLC3 cells appear to contain numerous autolysosomes containing autophagosome-like structures (Figure 3B, top right and bottom images). This observation agrees with data in Figure 3A (top graph) showing alteration in autophagy 3 h after gefitinib treatment. In SKBR3 cells, the response to gefitinib was similar to that found in MCF7-GFPLC3 cells and the results show a concentration-dependent rise in the average MDC TOA/cell up to 3 h treatment with gefitinib (Figure 3C). 

To confirm the imaging data showing a rise in the autophagosomal compartment, we performed Western blot analysis of soluble LC3-I and lipidated LC3-II levels in cells treated for 3 to 4 h with gefitinib. LC3-II, a cleaved and lipid-bound form of the LC3 protein, is an integral part of the autophagosomal membrane and serves as a marker of autophagosomes [35]. In gefitinib-sensitive SKBR3 and BT474 cells 3 h after adding gefitinib a concentration-dependent increase in LC3-II was observed starting at 1.3 µM and 0.6 µM, respectively (Figure 3D, top gels). In the gefitinib-insensitive MCF7-GFPLC3 cells treated for 4 h with gefitinib, the increase in LC3-II levels was evident at concentrations greater than 0.6 µM (Figure 3D, top gel).

In order to determine whether LC3-II accumulation is associated with gefitinib-mediated EGFR inhibition, Western blot analysis of EGFR and downstream signaling proteins within the MAPK and PI3K/AKT pathways was completed. After 3 h a decrease in phosphorylation (P) of EGFR, AKT, ERK1/2 and the mechanistic target of rapamycin (mTOR) substrates p70 ribosomal S6 kinase (p70S6K) and its downstream target S6 ribosomal protein (S6) as well as eukaryotic translation initiation factor 4E-binding protein-1 (4E-BP1) could be observed in SKBR3 and BT474 cells (Figure 3D, left and middle panels, respectively). Phosphorylation of these proteins was inhibited at therapeutically relevant concentrations of gefitinib (up to 1.3 µM). In gefitinib-insensitive MCF7-GFPLC3 cells, gefitinib-mediated changes in P-EGFR were hardly detectable and caused only a modest decrease in P-AKT and P-ERK levels (Figure 3D, right panels). Furthermore, P-p70S6K, P-S6 and P-4E-BP1 levels remained unchanged (Figure 3D, right panels) suggesting that mTOR complex 1 (mTORC1) signaling was not inhibited. Overall, the results presented in Figure 3 show that gefitinib-induced changes in EGFR signaling were cell-type specific and correlated with increased levels of the LC3-II protein. 

### Gefitinib enhances autophagic flux

**Figure 4:**
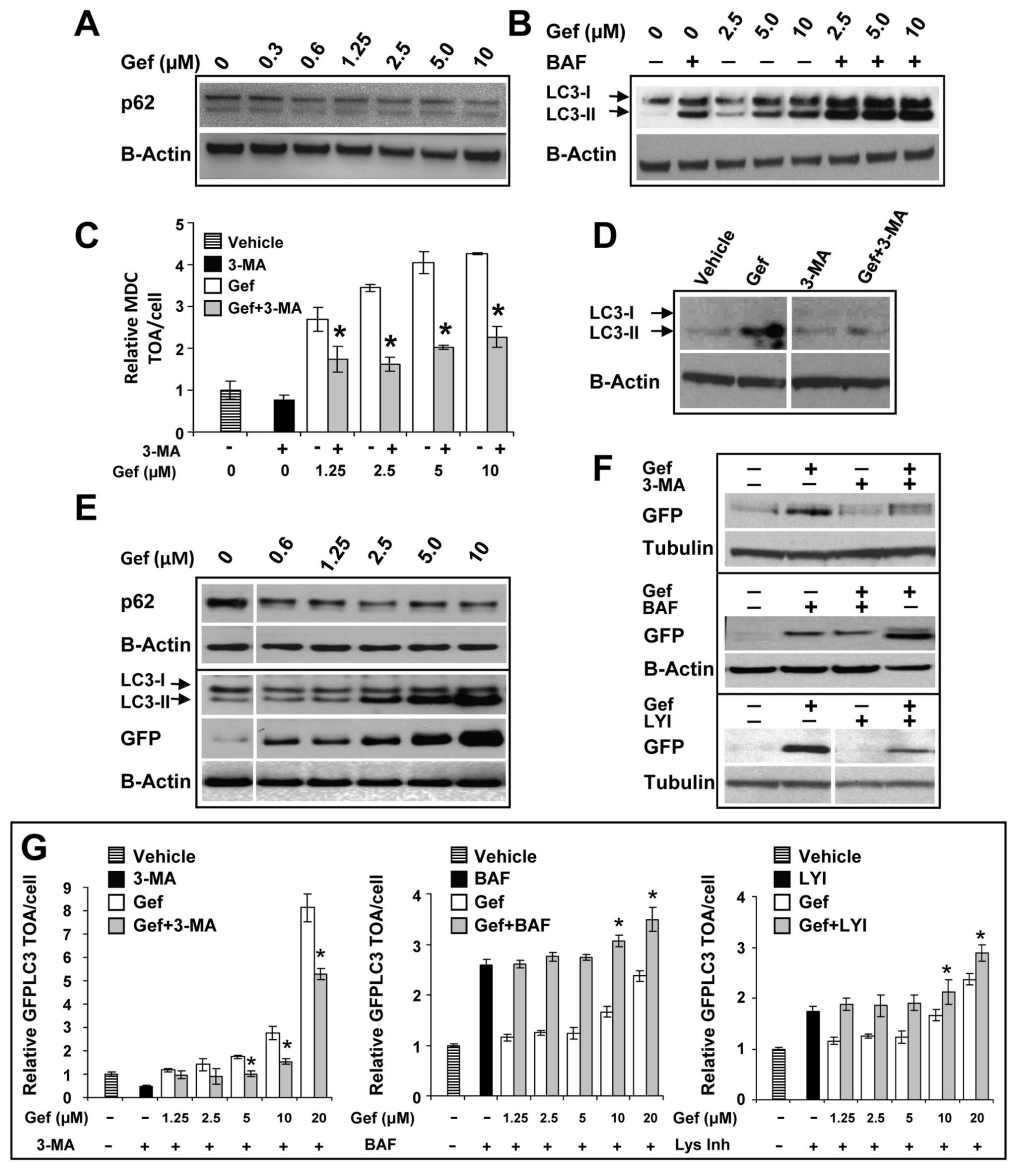
Gefitinib induces autophagic flux. (**A** - **D**) Autophagic flux assays performed in SKBR3 cells. (**A**) Western blot analysis of p62 expression in lysates derived from cells treated for 3 h with vehicle (0 µM gefitinib) or increasing concentrations of gefitinib (Gef) (**B**) Western blot analysis of LC3 levels in lysates derived from cells treated for 3 h with increasing concentrations of gefitinib in the absence or presence of 5 nM bafilomycin A1 (BAF). (**C**) HCA of TOA in cells treated for 72 h with increasing concentrations of gefitinib in the absence or presence of 10 mM 3-MA added for the last 3 h of treatment. The results are normalized to the vehicle control expressed as 1. Each bar represents the mean±SD from 3 replicate wells. Asterisks indicate statistically significant differences (p<0.05) between cells treated with gefitinib or 3-MA alone and cells treated with 3-MA in the presence of gefitinib. The results shown are representative of two experiments. (**D**) Western blot analysis of LC3-I and LC3-II levels in lysates derived from cells treated for 24 h with vehicle, 5 µM gefitinib, 5 mM 3-MA and the combination of gefitinib and 3-MA at the corresponding concentrations. Representative blots in (**A**), (**B**) and (**D**) are shown. (**E** - **G**) Autophagic flux assays performed in MCF7-GFPLC3 cells. (**E**) Western blot analysis of autophagic markers in lysates derived from cells treated for 18 h with increasing concentrations of gefitinib. Cleaved GFP is marked as GFP. (**F**) Western blot analysis of cleaved GFP levels in lysates derived from cells treated with vehicle or 10 µM gefitinib in the absence or presence of 10 mM 3-MA for 3 h (top panel), in the absence or presence of 50 nM bafilomycin A1 (BAF) for 24 h (middle panel), and in the absence or presence of 10 mg/ml lysosomal inhibitors (LYI; pepstatin A and E-64d) added for the last hour of treatment (bottom panel). Tubulin was used as loading control. Representative blots in (**E**) and (**F**) are shown. (**G**) HCA data showing the average GFPLC3 TOA/cell in MCF7-GFPLC3 cells treated for 3 h with increasing concentrations of gefitinib in the absence or presence of indicated autophagy inhibitors added for the last hour of treatment. The results are normalized to vehicle control expressed as 1. Each data point represents the mean±SD from 3 replicate wells and the results shown are representative of two experiments. 3-MA was used at 5 mM, bafilomycin A1 (BAF) was used at 5 nM and LYI were used at 10 µg/ml. Asterisks indicate statistically significant differences (p<0.05) between cells treated with gefitinib or autophagy inhibitors alone and cells treated with autophagy inhibitors in the presence of gefitinib.

 The results presented thus far provide compelling evidence that gefitinib alters autophagy in breast cancer cells. The increase in autophagosome numbers in cells could be a result of an intense stimulation of autophagy resulting in rapid formation of autophagosomes. Alternatively, this could be a reflection of reduced turnover in the autophagosomal compartment caused by impaired autophagosome-lysosome fusion and/or lysosomal function. In order to distinguish between these processes we investigated the status of autophagic flux in gefitinib-treated cells. First, the levels of p62 were measured. In SKBR3 cells Western blot analysis showed that levels of p62 (SQSTM1), a marker of autophagic flux [35], decreased in a concentration-dependent fashion after gefitinib treatment (Figure 4A). Further evidence that gefitinib elevates autophagic flux in SKBR3 cells was gained from experiments in which cells were treated with gefitinib in combination with bafilomycin A1, an inhibitor of the vacuolar ATPase (V-ATPase) required for lysosome acidification. Inhibition of V-ATPase causes increase in lysosomal pH, which in turn, renders lysosomes less effective in processing their cargo while also compromising their ability to fuse with autophagosomes [35,40]. The results of Western blot analysis presented in Figure 4B show that gefitinib or bafilomycin A1, when used alone, increased the lipidated form of LC3 (LC3-II). When combined, there were higher levels of LC3-II suggesting accumulation of autophagosomes in cells with hindered lysosomal function. This, in turn, suggests that gefitinib acts to increase autophagic flux. Further data confirming that gefitinib enhances autophagic flux in SKBR3 cells are shown in Figure 4C and D. Cells were treated with gefitinib in the presence of 3-MA, a class III PI3K (an essential enzyme required for the formation of early autophagosomes [35]) inhibitor added for the last 3 h of incubation. The HCA data show that in the presence of various concentrations of gefitinib, 3-MA significantly (p<0.05) decreased the average MDC TOA/cell (Figure 4C). Western blot analysis confirmed that 3-MA decreased levels of LC3-II in gefitinib-treated SKBR3 cells (Figure 4D), suggesting inhibition of autophagy. In MCF7-GFPLC3 cells, a reduction in p62 was noted after 18 h gefitinib treatment and this was associated with a concentration-dependent increase in LC3-II levels (Figure 4E). Autophagic flux was also confirmed in these cells with a functional assay monitoring proteolysis of GFPLC3 [35]. Following fusion of autophagosomes with lysosomes, the acidic hydrolases degrade the autophagosome and its content, including LC3. The GFP moiety of GFPLC3 is more resistant to proteolysis and remains intact for extended periods of time allowing for detection of cleaved GFP protein by Western blot analysis [35]. As shown in Figure 4E, concentration-dependent increases in proteolytic degradation of GFPLC3 are apparent in gefitinib-treated cells. The levels of cleaved GFP were decreased by 3-MA, bafilomycin A1 and the pepstatin A and E-64d (lysosomal inhibitors) compared to gefitinib treatment alone (Figure 4F). The HCA data confirmed that in the presence of gefitinib, 3-MA reduced redistribution of cytoplasmic GFPLC3 into puncta in MCF7-GFPLC3 cells (Figure 4G, left graph) and that bafilomycin A1 as well as lysosomal inhibitors increased the average GFPLC3 TOA/cell (Figure 4G, middle and right graphs, respectively). Overall, these results suggest that gefitinib influences the processes that control formation of autophagic organelles resulting in increased levels of autophagic flux.

### EGFR silencing stimulates autophagy

**Figure 5:**
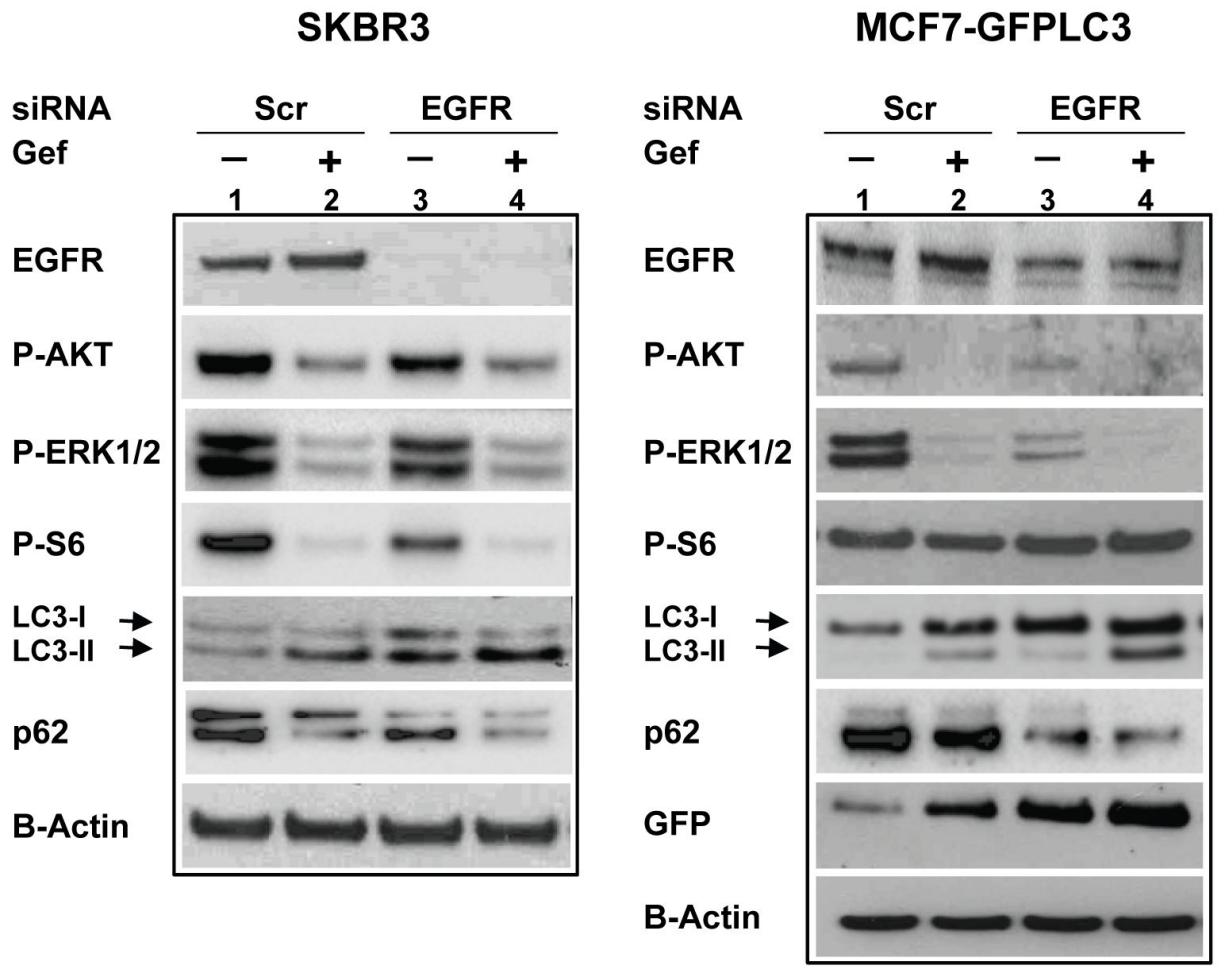
Effects of siRNA mediated EGFR silencing on downstream signaling and autophagy. (**A**) Following a transfection with EGFR siRNA, SKBR3 cells were treated for 72 h with vehicle or 5 µM gefitinib. (**B**) Following a double knockdown with the EGFR siRNA MCF7-GFPLC3 cells were treated with vehicle or 4 µM gefitinib for 24 h. Representative experiments are shown.

If gefitinib induces autophagy by targeting EGFR activity, then it is expected that EGFR downregulation would also result in autophagy induction. To test this, we performed siRNA-mediated EGFR silencing in SKBR3 and MCF7-GFPLC3 cells. Knockdown of EGFR resulted in decreased levels of EGFR mRNA in SKBR3 and MCF7-GFPLC3 cells (0.16±0.02 and 0.19±0.04, respectively, relative to non-silencing scrambled siRNA expressed as 1; Figure S3A). Western blot analysis of lysates derived from SKBR3 cells transfected with a scrambled and EGFR specific siRNA showed an effective downregulation of the EGFR protein levels (Figure 5). Despite using a double knockdown procedure to silence EGFR in MCF7-GFPLC3 cells, only a partial reduction in EGFR protein was attained (Figure 5). Functionality of the EGFR knockdown was confirmed by reduced levels of activated AKT and ERK1/2 in both SKBR3 and MCF7-GFPLC3 cells (lanes 1 and 3). Activity of the mTOR pathway, as shown by levels of activated S6, was reduced only in SKBR3 cells and not in MCF7-GFPLC3 cells, a result consistent with insensitivity of mTORC1 to gefitinib in these cells, and with data in Figure 3D. However, EGFR knockdown did not preclude gefitinib-mediated downregulation of its downstream targets (lanes 3 and 4). Changes in autophagy upon EGFR knockdown were associated with increased LC3-I/II, decreased p62 levels and higher levels of cleaved GFP (MCF7-GFPLC3) relative to scrambled siRNA, suggesting an increase in autophagic flux in the EGFR-reduced background (lanes 1 and 3). Still, EGFR knockdown did not prevent gefitinib-mediated autophagy in SKBR3 or MCF7-GFPLC3 cells as shown by increased LC3-II and cleaved GFP (MCF7-GFPLC3) as well as decreased p62 levels, when compared to vehicle-treated cells (lanes 3 and 4). These data suggest that the EGFR knockdown achieved in our experiments increased autophagic flux but poorly prevented gefitinib-induced autophagy.

### Effects of BECN1 and ATG7 silencing on autophagy and cell viability

**Figure 6:**
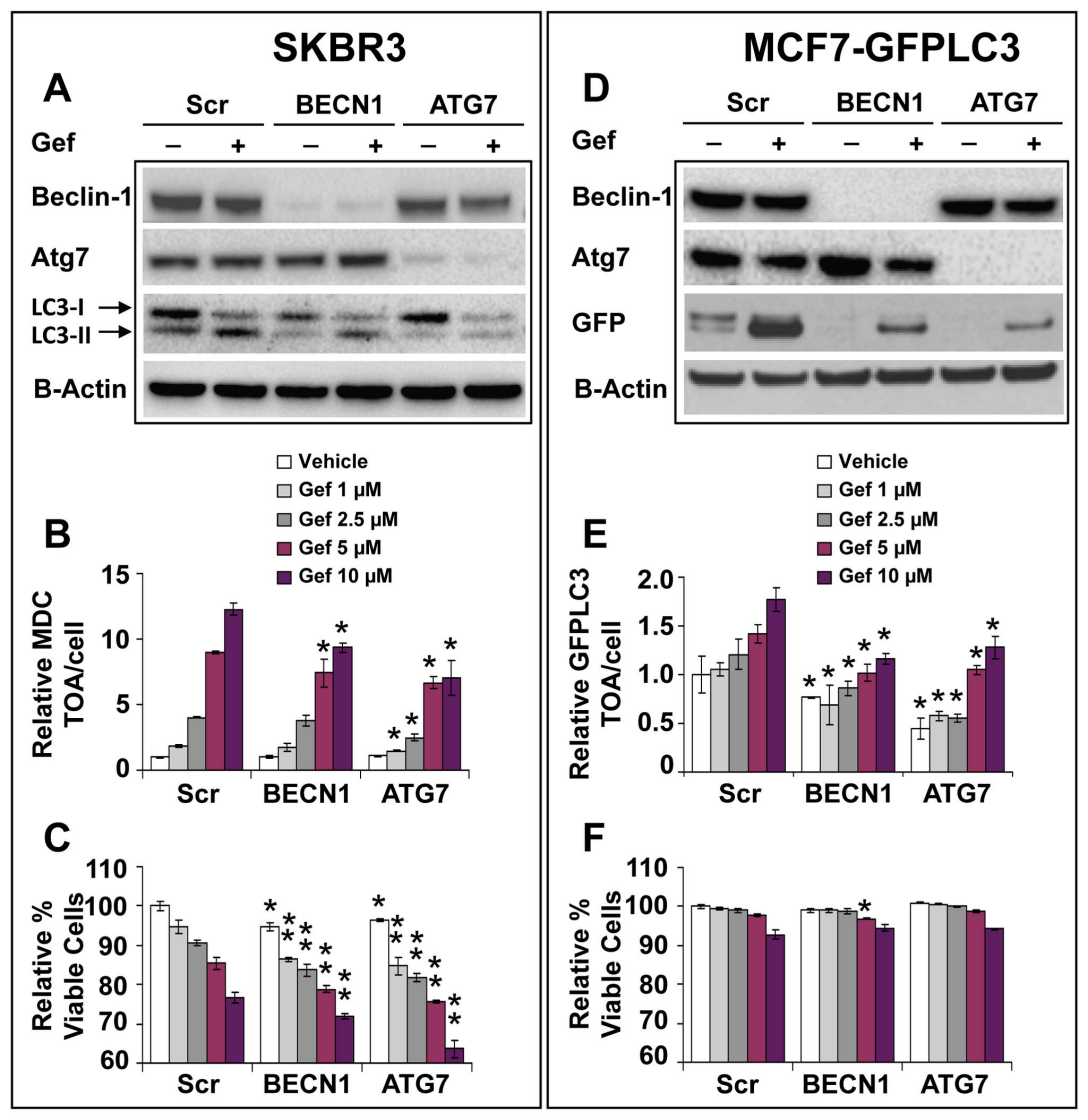
Effects of BECN1 or ATG7 knockdown on autophagy and viability in SKBR3 and MCF7-GFPLC3 cells. Following a knockdown with the indicated siRNA, cells were treated with vehicle or gefitinib (Gef) for 72 h. (**A** and **D**) Validation of BECN1 and ATG7 knockdown at the protein levels and analysis of LC3 and cleaved GFP by Western blotting. HCA analysis of TOA (**B** and **E**) and viability (**C** and **F**) in SKBR3 and MCF7-GFPLC3 cells following BECN1 or ATG7 knockdown (mean±SD, n = 3 wells). The average TOA and % viability are normalized to scrambled siRNA pretreated and vehicle-treated cells expressed as 1 or 100%, respectively. Cell viability was calculated as the proportion of viable cells in the total cell population. A single asterisk in (**B**), (**E**) and (**F**) indicates a statistically significant difference (p<0.05) between cells transfected with BECN1 or ATG7 and cells transfected with a scrambled non-silencing siRNA for the corresponding gefitinib concentration. A double asterisk in (**C**) indicates a statistically significant difference (p<0.05) between cells with knockdown genes and cells transfected with a scrambled non-silencing siRNA for a corresponding gefitinib concentration in addition to a statistically significant difference when compared to the vehicle-treated cells within each indicated knockdown.

Autophagy is a process tightly regulated by the *ATG* genes. *BECN1* (*ATG6*) is the prominent regulator involved in early autophagosome formation [12,35,41], and *ATG7* codes for a noncanonical, homodimeric E1 enzyme that takes part in a multistep process of LC3-I lipidation resulting in LC3-II-phosphatidylethanolamine (PE) essential for binding to autophagosome membranes [12,42]. We examined the effects of BECN1 and ATG7 siRNA in gefitinib-sensitive SKBR3 and gefitinib-insensitive MCF7-GFPLC3 cells treated with vehicle or gefitinib and the results are summarized in Figure 6. To recall, following a knockdown with the indicated siRNA, the qRT-PCR results showed that after 72 h mRNA expression relative to the non-silencing scrambled control siRNA expressed as 1 was 0.07±0.02 for BECN1 and 0.24±0.02 for ATG7 in SKBR3 cells and 0.006±0.001 and 0.25±0.03 for BECN1 and ATG7, respectively, in MCF7-GFPLC3 cells (Figure S3B). Western blot analysis confirmed the effective downregulation of BECN1 and ATG7 at the protein levels following siRNA treatment in SKBR3 and MCF7-GFPLC3 cells (Figure 6A and D). Inhibition of autophagy following knockdown of BECN1 and ATG7 was confirmed by lower levels of LC3-II or cleaved GFP relative to the scrambled siRNA, in SKBR3 and MCF7-GFPLC3 cells, respectively, in the absence and presence of gefitinib (Figure 6A and D). In support, HCA data showed that 72 h treatment with gefitinib resulted in a significant (p<0.05) reduction in MDC-positive (SKBR3) or GFPLC3-positive (MCF7-GFPLC3) organelles in cells pretreated with BECN1 or ATG7 siRNA compared to pretreatment with the non-silencing scrambled siRNA (Figure 6B and E). Additionally in MCF7-GFPLC3 cells, BECN1 or ATG7 knockdown caused a significant (p<0.05) reduction in the basal level of GFPLC3-positive organelles (Figure 6 E). HCA data also showed that reduction in autophagic organelle content in SKBR3 cells pretreated with BECN1 and ATG7 siRNA was associated with loss of cell viability which could be further decreased by co-treatment with gefitinib (72 h) (Figure 6C; p<0.05). In contrast, in MCF7-GFPLC3 cells, BECN1 or ATG7 siRNA on their own did not reduce viability and caused only negligible effects after 72 h treatment with gefitinib (Figure 6F). Longer exposure to BECN1 and ATG7 siRNA achieved by a double knockdown procedure over 96 h to minimize the expression of the corresponding proteins also did not affect viability of the vehicle or gefitinib-treated MCF7-GFPLC3 cells compared to non-silencing siRNA pretreated cells (data not shown). These results suggest that siRNA-mediated inhibition of early-stage autophagy is cytoprotective in SKBR3 cells but not in MCF7-GFPLC3 cells.

### Effects of pharmacological inhibition of gefitinib-induced autophagic flux in gefitinib-sensitive and -insensitive cells

**Figure 7:**
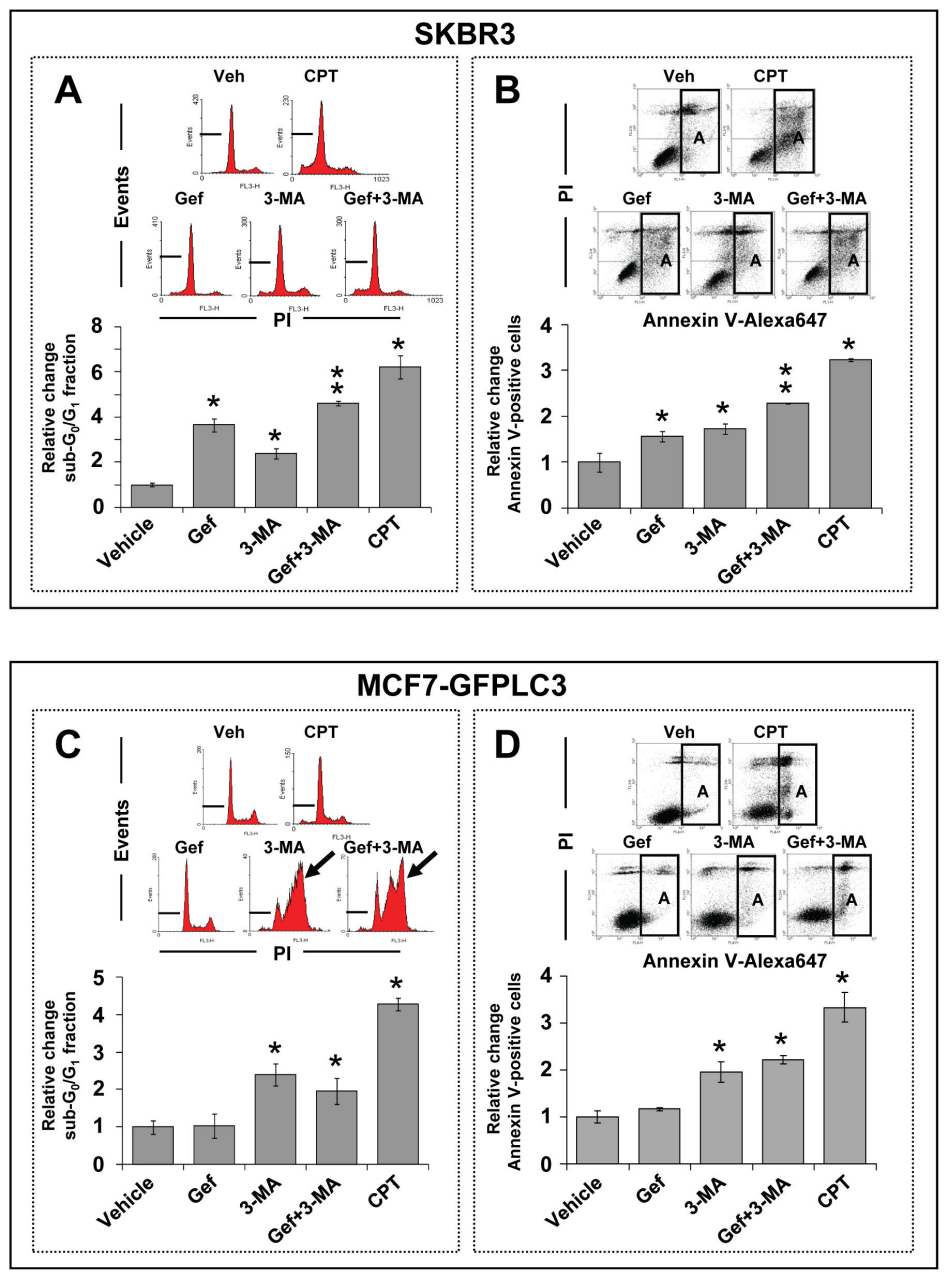
3-MA sensitized SKBR3 but not MCF7-GFPLC3 cells to cell death in the presence of gefitinib. Flow cytometric analysis of apoptosis in SKBR3 (**A** and **B**) and MCF7-GFPLC3 (**C** and **D**) cells treated for 72 h with vehicle or 10 µM gefitinib (Gef), in the absence or presence of 5 mM 3-MA. Camptothecin (CPT) at 5 µM was used as an inducer of apoptosis (positive control). (**A** and **C**) Analysis of the sub-G_0_/G_1_ apoptotic cell fraction. The inserted histograms show representative DNA profiles of cells treated with the indicated agents where a sub-G_0_/G_1_ cell fraction is indicated with a marker. Arrows in (**C**) indicate S-G_2_/M cell cycle block. (**B** and **D**) Analysis of apoptosis in cells stained with Annexin V-Alexa647 and PI. The inserted representative dot plots show distribution of cell populations treated with the indicated agents where apoptotic Annexin V-positive cells are marked as “A” in a rectangular region. Bar graphs represent the data (mean±SD) from 3 independently stained samples and show fold change relative to the vehicle-treated controls expressed as 1. Asterisks indicate a statistically significant difference (p<0.05) between cells treated with the vehicle and indicated agents. A double asterisk indicates a statistically significant difference (p<0.05) between cells treated with gefitinib in the presence of 3-MA and single-agent treated cells in addition to a statistically significant difference when compared to the vehicle-treated cells. Representative experiments are shown.

 Next, we investigated whether pharmacological inhibitors of autophagy may improve efficacy of gefitinib in sensitive and insensitive breast cancer cells. Blocking the early steps of basal levels of autophagy in vehicle treated cells with 3-MA significantly (p<0.05) increased apoptosis in SKBR3 (Figure 7A and B) and MCF7-GFPLC3 (Figure 7C and D) cells after 72 h treatment. However, in the presence of gefitinib 3-MA augmented apoptosis only in SKBR3 cells but not in MCF7-GFPLC3 cells, even when MCF7-GFPLC3 cells were co-treated with gefitinib and 3-MA for 144 h (data not shown). Of interest, in MCF7-GFPLC3 cells, 3-MA alone or in combination with gefitinib caused an S and G_2_/M block (Figure 7C).

**Figure 8:**
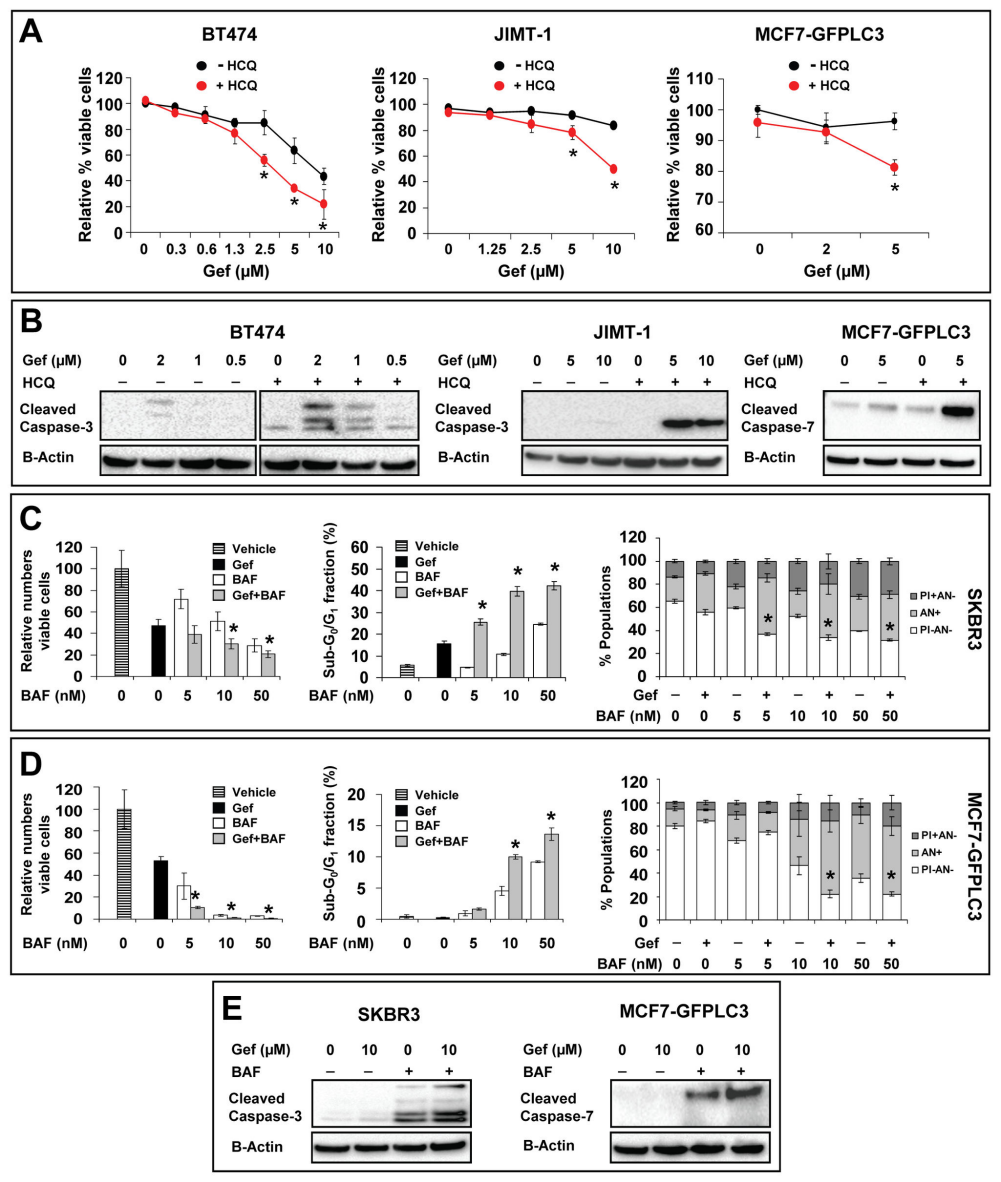
HCQ and bafilomycin A1 sensitize gefitinib-treated cells to cell death. (**A**) BT474, JIMT-1 and MCF7-GFPLC3 cells were treated for 24 h, 72 h and 168 h, respectively, with vehicle (0 µM gefitinib) or increasing concentrations of gefitinib (Gef) in the absence or presence of HCQ (20 µM). The HCA (BT474 and JIMT-1) and flow cytometric data (MCF7-GFPLC3) are expressed as percentage of viable ETH- or PI-excluding cells, respectively, in the total population normalized to vehicle-treated cells expressed as 100%. (**B**) Western blot analysis of caspase activation in lysates derived from cells treated as indicated in (**A**). (**C**) SKBR3 cells were treated for 72 h and (**D**) MCF7-GFPLC3 cells were treated for 120 h with vehicle, 10 µM gefitinib (Gef), increasing concentrations of bafilomycin A1 (BAF) and the combination of gefitinib and bafilomycin A1 (Gef+BAF). Left graphs: absolute numbers of viable (trypan blue excluding) cells in cultures treated with the indicated agents relative to cultures treated with vehicle expressed as 100%. Middle graphs: flow cytometric analysis of the sub-G_0_/G_1_ cell fraction in cells treated with the indicated agents (mean±SD, n = 3). Right graphs: flow cytometric analysis of apoptosis and viability based on Annexin V-Alexa647 and PI staining. PI+AN-: necrotic cells; AN+: apoptotic cells; PI-AN-: viable cells. Each data point in (**A**), (**C**) and (**D**) represents a mean±SD from 3 replicate samples. Asterisks indicate a significant difference (p<0.05) between cells treated with gefitinib and the combination of gefitinib and HCQ in the same category. The results shown are representative of 2 experiments for each cell type. (**E**) Western blot analysis of caspase activation in lysates derived from SKBR3 and MCF7-GFPLC3 cells treated for 72 h and 120 h, respectively, with vehicle or gefitinib (Gef) in the absence or presence of 10 nM bafilomycin A1 (BAF).

In contrast, HCQ and bafilomycin A1 that inhibit autophagy at a late stage were effective in sensitizing SKBR3 and MCF7-GFPLC3 cells to gefitinib. HCQ is a weak base which accumulates in lysosomes and increases intralysosomal pH [43]. This, in turn, inhibits degradation of the lysosomal cargo and disables completion of the autophagic process [35,43]. Figure 8A presents viability data for gefitinib-sensitive (BT474) and gefitinib-insensitive cells (JIMT-1 and MCF7-GFPLC3) treated for 24 h, 72 h or 168 h, respectively, with gefitinib in the absence or presence of HCQ. The different time points for each cell type were selected to reflect the differences in gefitinib sensitivity of these cell lines. The data show that HCQ in combination with gefitinib (2.5 - 10 µM) significantly decreases (p<0.05) viability in BT474 cells when compared to cells treated with gefitinib alone (Figure 8A, left graph). In JIMT-1 cells treated with HCQ there was also a statistically significant (p<0.05) drop in viability when gefitinib was present at 5 - 10 µM (Figure 8A, middle graph). In MCF7-GFPLC3 cells (Figure 8A, right graph) the combination of 5 µM gefitinib with HCQ decreased viability, when compared to gefitinib treatment alone, but this was only apparent after 168 h. Our data also show that treatment with the gefitinib and HCQ combination led to increased activity of caspase-3 in BT474 and JIMT-1 cells and caspase-7 in MCF7-GFPLC3 (caspase-3 deficient) cells relative to either treatment alone (Figure 8B). This suggests involvement of apoptosis in gefitinib and HCQ co-treated cells. The effects of autophagy inhibition at the late stage were also confirmed using bafilomycin A1. The data in Figure 8C (left graph) show that 72 h treatment with the combination of gefitinib and bafilomycin A1 (used at 10 and 50 nM) decreased the absolute number of viable cells in SKBR3 culture more effectively than either agent alone (p<0.05). This was accompanied by a bafilomycin A1 dose-dependent increase in apoptosis where statistically significant (p<0.05) increases in the sub-G_0_/G_1_ fraction and Annexin V-positive cells were noted when the drugs were used in combination relative to the effects of gefitinib or bafilomycin A1 alone (Figure 8C, middle and right graphs). In MCF7-GFPLC3 cells, the cytotoxic effects of the gefitinib and bafilomycin A1 combination were apparent much later, thus the data shown in Figure 8D were obtained after 120 h treatment. These data show that when bafilomycin A1 was used in combination with gefitinib, there was a statistically significant (p<0.05) decrease in the absolute number of viable cells (Figure 8D, left graph) and a parallel increase in apoptosis, relative to effects of gefitinib or bafilomycin A1 alone (Figure 8D, middle and right graphs). Increased levels of apoptosis in SKBR3 and MCF7-GFPLC3 cells treated with the gefitinib and bafilomycin A1 combination were confirmed by Western blot analysis demonstrating higher activity of caspases in cells treated with the combination relative to the single agents (Figure 8E). Taken as a whole, these data suggest that lysosomal impairment by HCQ or bafilomycin A1 improves efficacy of gefitinib and this could be attributed, at least in part, to caspase-dependent apoptotic cell death.

### HCQ in combination with gefitinib enhances therapeutic response of JIMT-1 tumors

**Figure 9:**
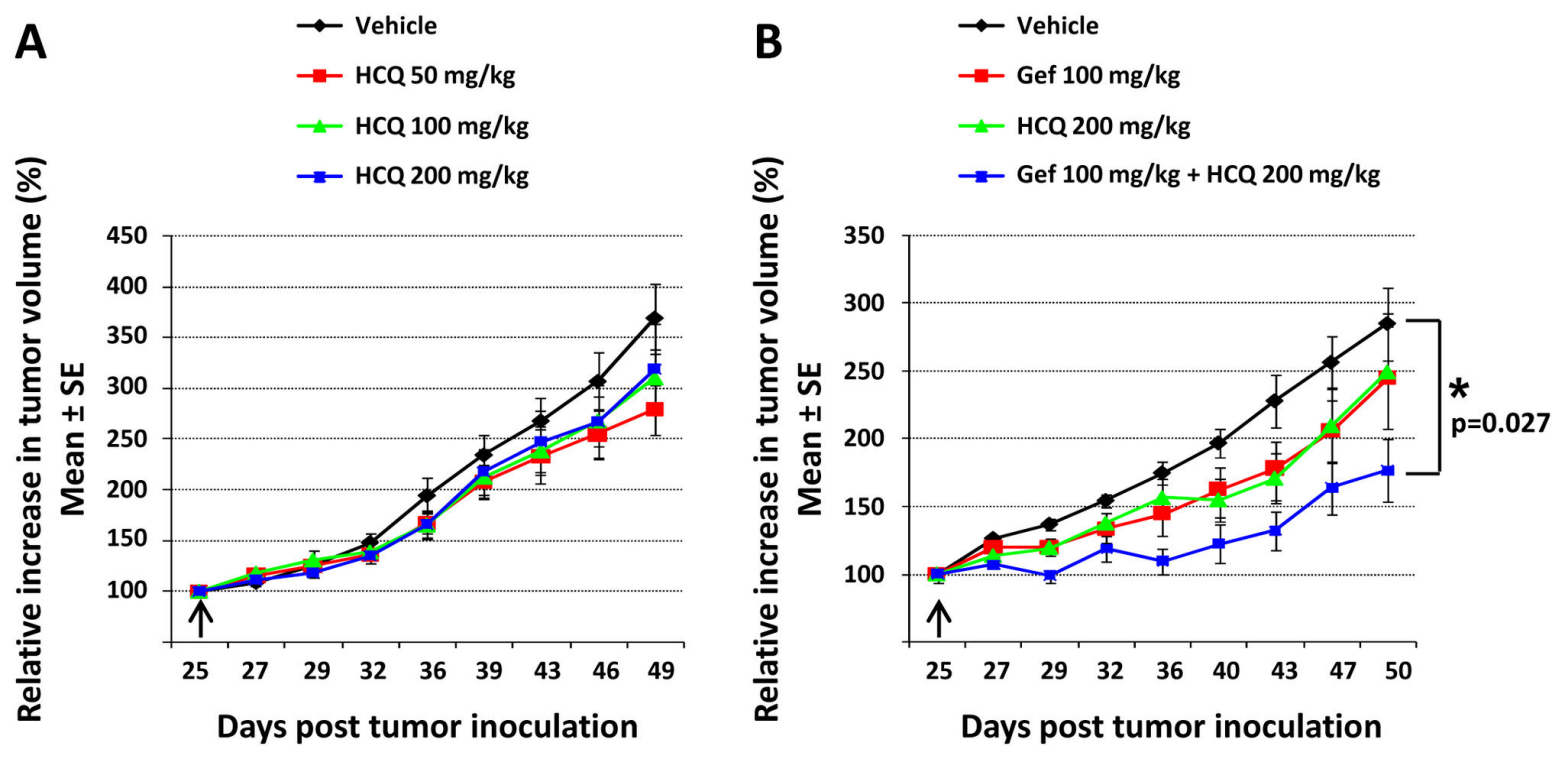
*In* *vivo* efficacy of HCQ alone and in combination with gefitinib. Mice bearing JIMT-1 tumors were treated (oral gavage) with the indicated agents (n = 6 mice per treatment group). Increase in tumor volume was plotted relative to the volume on the first day of treatment (indicated by arrows) expressed as 100%. (**A**) Efficacy of HCQ used at different doses. (**B**) Efficacy of the gefitinib and HCQ combination. An asterisk represents a significant (p<0.05) difference between vehicle and combination treated animals on the last day of treatment.

The *in vitro* data presented in Figure 8 suggest that inhibition of gefitinib-induced autophagy by lysosomotrophic agents HCQ and bafilomycin A1 sensitizes breast cancer cell lines to cell death, irrespective of gefitinib sensitivity status. To test the efficacy of this combination *in vivo*, animals bearing established gefitinib-insensitive JIMT-1 tumors were treated with gefitinib, HCQ or a combination of the two drugs, each delivered as oral gavage. The results summarized in Figure 9A show that HCQ dosed for a period of 25 days at 50 to 200 mg/kg did not significantly (p>0.05) reduce JIMT-1 tumor volume relative to vehicle, as measured on the last day of treatment. HCQ was well tolerated at all doses, thus, the highest tested dose of HCQ (200 mg/kg) was chosen for the combination study. A dose of 100 mg/kg gefitinib was selected based on previously published results [36]. As shown in Figure 9B, treatment of JIMT-1 xenografts for 26 days with either gefitinib or HCQ alone reduced average tumor volume by only 22% and 19% respectively, compared to tumors in the vehicle-treated animals (p>0.05). Notably, when gefitinib was used in combination with HCQ there was a significant (p<0.05) 58% reduction in tumor volume compared to vehicle-treated controls (Figure 9B). Even though tumor volume in animals treated with the combination was on average 47% and 49% lower relative to gefitinib and HCQ monotherapies, respectively, statistical significance was not achieved (p>0.05). Detailed animal health monitoring data (not reported here) suggested that gefitinib and HCQ were well tolerated regardless of whether the drugs were used alone or in combination. The reported body weight loss in animals did not exceed a nadir of 10±4% (mean±SD). These results demonstrate that the combination of gefitinib with the late-stage autophagy inhibitor HCQ was safe and effective in delaying growth of gefitinib-insensitive JIMT-1 tumors. 

### Gefitinib-induced autophagic flux is a reversible process

**Figure 10:**
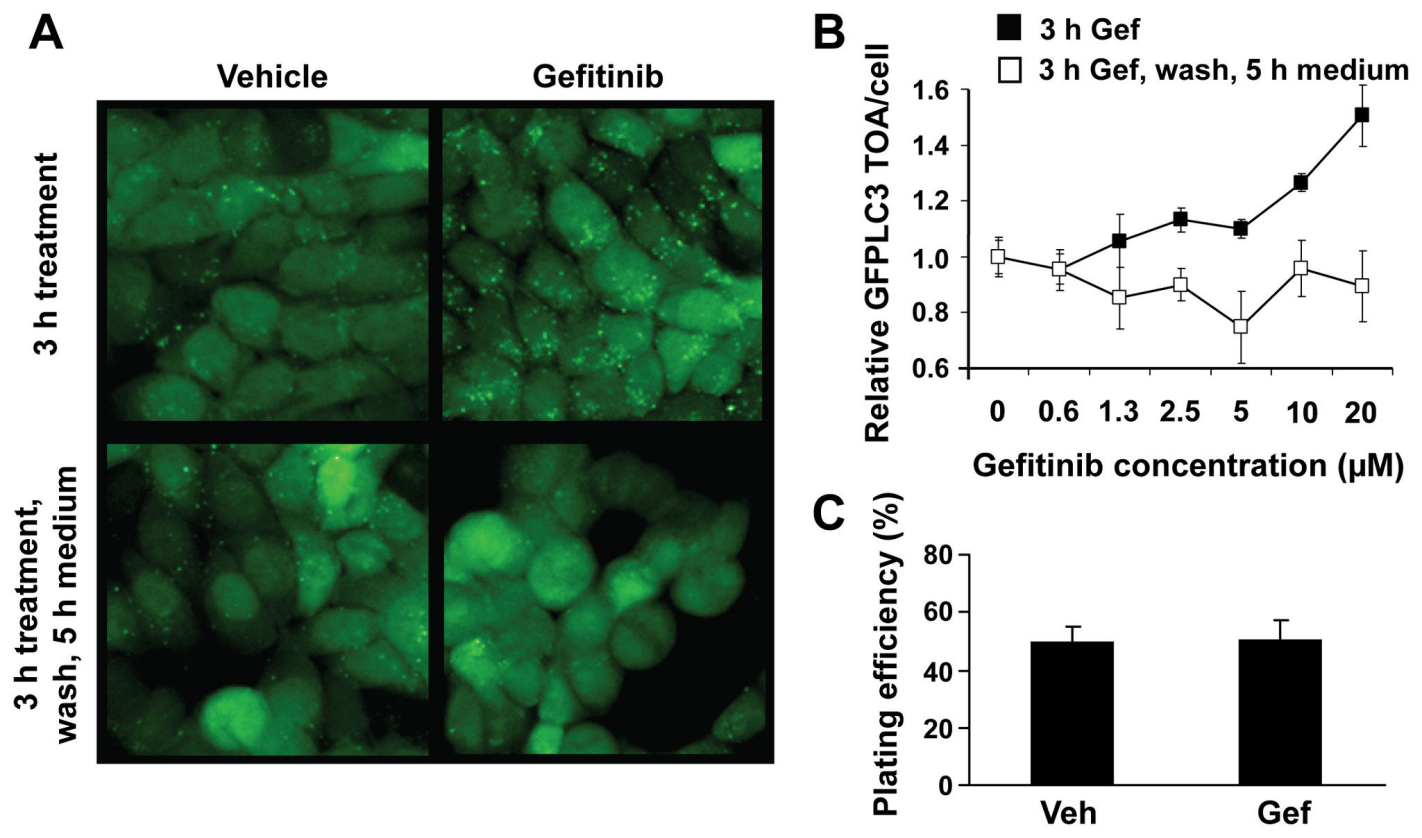
Gefitinib-induced autophagy is a reversible process. (**A**) Representative images of MCF7-GFPLC3 cells treated for 3 h with vehicle or 10 µM gefitinib (top images) obtained with IN Cell 1000 Analyzer. After the indicated treatment, cell cultures were washed twice with medium and then incubated in the absence of drug for the following 5 h (bottom images). GFPLC3-labeled autophagosomes appear as green fluorescent puncta in cellular cytoplasm. (**B**) MCF7-GFPLC3 cells were treated for 3 h with vehicle (0 µM) or increasing concentrations of gefitinib and washed as described in (**A**). Quantitation of GFPLC3 TOA/cell by HCA in MCF7-GFPLC3 cells; the data shown are normalized to vehicle-treated cells expressed as 1 (mean±SD, n = 3 replicate wells). (**C**) Clonogenic assay performed in MCF7-GFPLC3 cells treated for 3 h with vehicle (Veh) or 10 µM gefitinib (Gef) followed by drug removal and incubation in media for 17 days. The results are expressed as % plating efficiency representing the proportion of cells that are able to form colonies. Bar graphs represent colony count from 3 wells (mean±SD). A representative experiment is shown.

 The gefitinib concentrations used in the studies described here include those in a clinically relevant range (~ 1 µM), but in some cases are beyond that achievable in the plasma of patients. It is recognized, however, that gefitinib concentrations in the blood compartment and the tumor tissue change as a function of time and peak drug levels can be high for a short time frame [39,44]. Unlike mechanisms that result in selection of treatment-resistant cell subpopulations, cytoprotective responses, such as stress-induced increases in autophagic flux, likely arise when the cancer cells are first subjected to a stress and then are reversed when the stress is removed. This could represent the clinical scenario where target cell populations are transiently exposed to higher levels of drug during the times when peak plasma concentrations have been reached. Perhaps more importantly, if the cytoprotective responses are reversible then this will have an important impact on how autophagy inhibiting agents are used clinically. Thus, we assessed whether gefitinib-induced changes in autophagic flux are reversible. The images presented in Figure 10A show that GFPLC3-labeled organelles accumulated over a three hour time frame in MCF7-GFPLC3 cells exposed to gefitinib (upper panels). After gefitinib is removed, however, the level of GFPLC3-labeled organelles reverts back to the background levels (Figure 10A, lower panels). Quantitation of the average GFPLC3 TOA/cell performed with the HCA methods shows the reversibility of autophagosome formation over a broad spectrum of gefitinib concentrations (Figure 10B). Furthermore, clonogenic data show that cell viability after 3 h treatment with gefitinib followed by drug removal was not affected, suggesting the absence of long term effects on cell growth by transient exposure to gefitinib (Figure 10C). These data indicate that gefitinib-induced autophagy is a transient reversible response. 

## Discussion

Targeted therapies such as trastuzumab (TZ (Herceptin®)) and lapatinib are approved for patients with HER2-positive breast cancer [45,46] but intrinsic and acquired insensitivity presents an important clinical problem [47-50]. In advanced breast cancers treated sequentially with numerous chemotherapies, the selection of “the fittest” cells able to survive therapeutic stress results in decreased genetic stability and induction of long term adaptations that support development of drug resistance [51,52]. Thus, strategies to address resistance before it arises may be more beneficial than attempting to treat refractory cancer after it acquires resistance mechanisms. For this reason our team’s efforts have been focused on the development of therapies addressing an understudied problem of early cytoprotective responses which arise when the cancer cells are initially exposed to therapeutic stress. 

Using HCA, TEM and molecular analysis we demonstrated in this report that gefitinib, an EGFR TKI, stimulates the appearance of autophagy-associated organelles in phenotypically diverse breast cancer cells. Alterations in autophagy were visible as early as 45 min after addition of gefitinib, a time-frame that is comparable to other autophagy inducing agents [40]. The relocation of GFPLC3 to autophagosomes and accumulation of autophagy-associated MDC and LTR-labeled organelles reached considerable levels within 3 h of gefitinib addition, and it was accompanied by decreasing distances between these organelles. The appearance of autophagic organelles in gefitinib-treated cells correlated with downregulation of EGFR, AKT, ERK1/2 and mTORC1 signaling in gefitinib-sensitive SKBR3 and BT474 cells. In gefitinib-insensitive MCF7-GFPLC3 cells, gefitinib-mediated effects on EGFR, AKT and ERK1/2 signaling were modest. This could be explained at least partially by low expression of EGFR and also HER2 which can dictate gefitinib’s activity in breast cancer cells [7,8]. Interestingly, phosphorylation of p70S6K, S6 and 4E-BP1 remained unchanged in MCF7-GFPLC3 cells, suggesting that gefitinib-induced autophagy may be mTORC1-independent in this cell line. In support of this, a wide variety of growth factors and cytokines have been shown to regulate autophagy through diverse signaling pathways that converge on the type III PI3K but do not require mTORC1 [53]. 

Further, we determined that greater autophagosome content in gefitinib-treated cells is a result of increased autophagic flux and not accumulation of autophagosomes due to inhibition of autophagy [35,40]. This was supported by data showing accumulation of GFPLC3- and MDC-labeled organelles accompanied by a rise in LC3-II and cleaved GFP levels and a concomitant decrease in p62 in gefitinib-treated cells. In addition, 3-MA brought about a reduction, while bafilomycin A1 and lysosomal inhibitors caused further accumulation of autophagic organelles in gefitinib-treated cells. The appearance of autophagosomes and autolysosomes containing cellular material in different stages of degradation was also confirmed by TEM data. These data suggest that gefitinib induces autophagy in gefitinib-sensitive and -insensitive breast cancer cells. To the best of our knowledge, a thorough investigation of gefitinib-induced autophagy has not been previously conducted in breast cancer. However, other targeted agents such as TZ, lapatinib and cetuximab have been shown to induce autophagy in breast cancer and this process contributed to development of resistance [17,19,21]. Inhibition of the EGFR tyrosine kinase by gefitinib and erlotinib has also been shown to induce autophagy in non-small-cell lung cancer [18,54] and blocking the EGFR receptor function with cetuximab induced autophagy in human vulvar squamous carcinoma, colorectal adenocarcinoma and head and neck cancer cells [21]. Hence, our results add to a growing body of evidence showing that agents targeting members of the EGFR receptor family induce autophagic responses.

To investigate if gefitinib-mediated autophagy is a consequence of EGFR-specific kinase inhibition, we targeted EGFR by siRNA. Our data showed that silencing EGFR increased autophagy levels in SKBR3 and MCF7-GFPLC3 cells, thus confirming its involvement in autophagy. When considering these data it is important to be aware of the kinase-independent (gefitinib-insensitive) function of EGFR that has been shown to support cell survival in the presence of therapeutic agents and TKI by maintaining the basal intracellular glucose levels [55]. In the absence of this kinase-independent function, cells were found to undergo autophagy to compensate for energy losses [55]. Thus, knockdown of EGFR may contribute to induction of autophagy through reduction in EGFR tyrosine kinase activity and in total EGFR levels. However, in the presence of gefitinib, autophagy was further elevated in EGFR knockdown cells, as judged by increased LC3-II and cleaved GFP and decreased p62 levels. Similar findings were reported by Han et al. who showed that EGFR silencing resulted in increased LC3-II expression in gefitinib-treated lung cancer cells [18]. Interestingly, autophagy induction in MCF7-GFPLC3 cells by EGFR siRNA or gefitinib was not associated with changes in S6 phosphorylation, providing additional evidence that autophagy may be regulated by an mTORC1-independent mechanism in this cell line. Unexpectedly, gefitinib was still able to reduce phosphorylation of AKT and ERK1/2 in EGFR knockdown cells. This could be due to incomplete knockdown of EGFR, such as in MCF7-GFPLC3, where residual EGFR tyrosine kinase activity may still be susceptible to gefitinib regulation. Alternatively, EGFR-independent effects of gefitinib such as inhibition of HER2 [7,8], or gefitinib’s secondary targets [11,56] could be contributing to changes in downstream signaling, especially when gefitinib is used at concentrations ≥ 1 µM as in our EGFR knockdown experiment. Thus, it is reasonable to assume that effects engendered by EGFR siRNA combined with gefitinib-mediated inhibition of AKT and ERK1/2 signaling in EGFR knockdown cells may have contributed to added increase in autophagy. 

Direct evidence supporting the fact that gefitinib is responsible for inducing autophagy comes from experiments showing that siRNA mediated silencing of BECN1 and ATG7 results in a significant decrease (p<0.05) of autophagic organelles in treated cells. However, inhibition of early-stage autophagy with BECN1 and ATG7 siRNA augmented cytotoxicity in the presence of gefitinib only in SKBR3 cells (p<0.05) with negligible effects in MCF7-GFPLC3 cells, suggesting that early-stage gefitinib-induced autophagy was cytoprotective in the former. Similarly, blocking early-stage autophagy with 3-MA augmented gefitinib’s cytotoxicity in SKBR3 but not in MCF7-GFPLC3 cells. These cell-type specific differences in the response to inhibition of early-stage gefitinib-induced autophagy could be related to a unique cross-talk between autophagy and apoptosis. The complex mechanisms involved in this cross-talk depend on the genetic make-up of cells and are not yet fully understood [57]. It could be speculated that this cross-talk antagonizes apoptotic pathways by engaging alternative survival mechanisms and decreases the likelihood of undergoing cell death following inhibition of early-stage autophagy in gefitinib-treated MCF7-GFPLC3 cells. Other studies have reported benefits of inhibiting autophagy at the late *versus* early stage in temozolomide and imatinib treated malignant glioma cells [58,59]. Likewise, our results show that inhibiting late-stage autophagy with HCQ and bafilomycin A1 is an effective strategy to increase cell death, involving apoptosis, in gefitinib-sensitive and -insensitive breast cancer cells. These results suggest that intact autophagic flux is critical for cell survival in the presence of elevated autophagy levels and support the cytoprotective role of autophagy in gefitinib-treated cells. Furthermore, we speculate that this cytoprotection may in part contribute to the maintenance of an innate resistance (insensitivity) to gefitinib in gefitinib-insensitive cells. However, it needs to be acknowledged that lysosomotropic agents may exert effects other than autophagy inhibition [60,61] that could also contribute to sensitization to gefitinib. It is also important to highlight that HCQ or bafilomycin A1 in the presence of gefitinib exerted cytotoxic effects when the latter was used at concentrations above 1 µM (the average achievable level of this drug in human plasma), thus off-target effects of gefitinib must be considered [7,8,11]. Still, a dose above 1 µM may be therapeutically relevant since gefitinib may accumulate in tumor tissue at concentrations much higher than measured in the plasma compartment [39]. More specifically, in breast cancer patients receiving a daily oral dose of 250 mg/day, gefitinib preferentially distributes from plasma into tumor tissue where it can reach levels 42 times higher than measured in plasma (mean levels of 16.7 µM) [39]. It is plausible that these high, localized concentrations of gefitinib can contribute to induction of autophagy in tumor tissue and that off-target effects of the drug will influence clinical response. 

Importantly, our report demonstrates *in vivo* that the combination of gefitinib with the autophagy inhibitor HCQ was more potent in inhibiting growth of gefitinib-insensitive JIMT-1 tumors than either monotherapy when compared to vehicle-treated controls. These data are consistent with our *in vitro* results showing sensitization of JIMT-1 cells to gefitinib in the presence of HCQ. While complete inhibition of JIMT-1 tumor growth was not achieved, this could be a consequence of our experimental design, in which gefitinib and HCQ were not used at maximum tolerated dose. Instead, we applied doses that on their own did not produce statistically significant (p>0.05) reduction in tumor volume, so any additional effect of the combination would be evident. Our results are encouraging considering that HCQ was dosed orally, as in the clinical setting. Future experiments will be required to demonstrate the effects of the combination containing higher doses of gefitinib and HCQ. Since insensitivity of breast cancers to EGFR targeted therapies presents a clinical challenge, it would be worthwhile to determine if gefitinib in combination with HCQ is broadly effective in the gefitinib-insensitive phenotype. Still, one should keep in mind that the sensitizing effects of HCQ *in vivo* in combination with chemotherapy may arise independently from its role as an autophagy inhibitor [43,62]. Nevertheless, our data add to a growing number of studies demonstrating a therapeutic gain of using HCQ in combination with various anti-cancer agents [29,31,43,63]. This approach is currently being tested in numerous clinical trials, including studies focused on breast cancer [31,32]. Notably, SKBR3 and MCF7-GFPLC3 cells contain monoallelic deletions of a tumor suppressor gene *BECN1* [64], a prominent regulator of early phagosome formation, observed in ~40% of human sporadic breast cancers [65]. Yet, our data suggest that gefitinib is still able to induce an intense autophagic flux in this genetic background and this may be advantageous when designing combination therapies with HCQ in the clinic.

Perhaps most interestingly, our *in vitro* data show that the intensity of gefitinib-induced autophagy depends on drug concentrations and that autophagy is reversible upon removal of the drug (see Figure 10). In patients, drug concentration will change over time and this is driven by the drug’s pharmacokinetics. Peak concentrations are followed by a decrease in drug level towards steady state (the desired goal of repeated dosing) [39,44]. We speculate that targeting autophagy at peak drug concentrations, where the early cytoprotective responses are expected, could yield better sensitization of cells to autophagy-promoting drugs. This would require that an autophagy inhibitor, such as HCQ, should be present in tumor tissue at a concentration sufficient to modulate the autophagic response during peak exposure to the autophagy-promoting drug. HCQ at the concentration used in our *in vitro* assays (20 µM) would be difficult to achieve in humans, as 1.5 to 3 µM were measured in plasma after repeated daily administration [62]. Therefore, developing new effective inhibitors of autophagy and utilizing drug delivery systems to optimize concentration of these agents at the tumor site should be beneficial. 

In conclusion, the results presented here suggest that treatment protocols based on gefitinib and other targeted drugs must consider their role as autophagy modulators. Although the scope of this study focuses on autophagy induced by gefitinib, it is anticipated that targeting early cytoprotective responses will be applicable to other cytoprotective mechanisms and other drugs. Inhibition of these cytoprotective responses within the context of breast cancer should be considered in the clinic when developing more effective drug combinations.

## Supporting Information

Figure S1 **Quantitation of autophagy-associated organelles by HCA methods in SKBR3 and MCF7-GFPLC3 cells treated with vehicle, gefitinib or tamoxifen.** (**A**) Representative images obtained with IN Cell 1000 Analyzer of SKBR3 cells treated for 48 h with vehicle or 10 μM gefitinib stained with lysotracker red (LTR) (red) or MDC (green) and counterstained with DRAQ5 (blue). Bottom graph: the average MDC TOA/cell (mean±SD, n = 6 replicate wells) obtained with HCA in vehicle (Veh) or gefitinib (Gef) treated cells. (**B**) Representative images of MCF7-GFPLC3 cells treated for 48 h with vehicle, 20 μM gefitinib or 10 μM tamoxifen stained with Hoechst 33342 (blue) and LTR (red). Green puncta represent GFPLC3-labeled autophagosomes, red puncta represent lysosomes and yellow puncta represent autolysosomes. Bottom graph: the average MDC and GFPLC3 TOA/cell (mean±SD, n = 6 replicate wells) obtained with HCA in vehicle (Veh), gefitinib (Gef) or tamoxifen (Tam) treated cells.
(TIF)

Figure S2 **The dynamics of autophagy-associated organelle formation in MCF7-GFPLC3 cells treated with gefitinib.** Representative images of MCF7-GFPLC3 cells treated with vehicle (0 µM gefitinib) or indicated gefitinib concentrations acquired with IN Cell 1000. GFPLC3 panel: the green background in the control cells represents the GFPLC3 protein which is diffusely spread throughout the cytoplasm. With time the GFPLC3 staining becomes more defined and GFPLC3-labeled organelles (green puncta) marking the location of autophagosome membrane associated LC3-II protein are observed in cells. LTR panel: images of MCF7-GFPLC3 cells stained with Hoechst 33342 (blue nuclei) and lysotracker red (LTR; red puncta). MDC panel: images of MCF7-GFPLC3 cells stained with DRAQ5 (blue) and MDC (green puncta) in the cellular cytoplasm. Images were pseudo-colored and overlaid using the Investigator software.
(TIF)

Figure S3 **Validation of siRNA-mediated knockdown by qRT-PCR.** (**A**) Levels of EGFR mRNA in SKBR3 cells harvested 72 h post knockdown and in MCF7-GFPLC3 cells harvested 48 h post double knockdown. (**B**) Levels of BECN1 and ATG7 mRNA in SKBR3 and MCF7-GFPLC3 cells harvested 72 h post knockdown. mRNA expression for each of the indicated genes in (**A**) and (**B**) is shown relative to the scrambled non-silencing siRNA control expressed as 1. Each data point represents a mean±SD from 3 replicate PCR samples.
(TIF)

## References

[1] Wiseman SM, Makretsov N, Nielsen TO, Gilks B, Yorida E et al. (2005) Coexpression of the type 1 growth factor receptor family members HER-1, HER-2, and HER-3 has a synergistic negative prognostic effect on breast carcinoma survival. Cancer 103: 1770-1777. doi:10.1002/cncr.20970. PubMed: 15770691. 15770691

[2] DiGiovanna MP, Stern DF, Edgerton SM, Whalen SG, Moore D 2nd et al. (2005) Relationship of epidermal growth factor receptor expression to ErbB-2 signaling activity and prognosis in breast cancer patients. J Clin Oncol 23: 1152-1160. doi:10.1200/JCO.2005.09.055. PubMed: 15718311. 15718311

[3] Rimawi MF, Shetty PB, Weiss HL, Schiff R, Osborne CK et al. (2010) Epidermal growth factor receptor expression in breast cancer association with biologic phenotype and clinical outcomes. Cancer 116: 1234-1242. doi:10.1002/cncr.24816. PubMed: 20082448. 20082448 PMC2829330

[4] Ritter CA, Perez-Torres M, Rinehart C, Guix M, Dugger T et al. (2007) Human breast cancer cells selected for resistance to trastuzumab in vivo overexpress epidermal growth factor receptor and ErbB ligands and remain dependent on the ErbB receptor network. Clin Cancer Res 13: 4909-4919. doi:10.1158/1078-0432.CCR-07-0701. PubMed: 17699871. 17699871

[5] Saxena R, Dwivedi A (2012) ErbB family receptor inhibitors as therapeutic agents in breast cancer: current status and future clinical perspective. Med Res Rev 32: 166-215. doi:10.1002/med.20209. PubMed: 22183797. 22183797

[6] Cohen MH, Williams GA, Sridhara R, Chen G, McGuinn WD Jr. et al. (2004) United States Food and Drug Administration Drug Approval summary: Gefitinib (ZD1839; Iressa) tablets. Clin Cancer Res 10: 1212-1218. doi:10.1158/1078-0432.CCR-03-0564. PubMed: 14977817. 14977817

[7] Moasser MM, Basso A, Averbuch SD, Rosen N (2001) The tyrosine kinase inhibitor ZD1839 ("Iressa") inhibits HER2-driven signaling and suppresses the growth of HER2-overexpressing tumor cells. Cancer Res 61: 7184-7188. PubMed: 11585753. 11585753

[8] Moulder SL, Yakes FM, Muthuswamy SK, Bianco R, Simpson JF et al. (2001) Epidermal growth factor receptor (HER1) tyrosine kinase inhibitor ZD1839 (Iressa) inhibits HER2/neu (erbB2)-overexpressing breast cancer cells in vitro and in vivo. Cancer Res 61: 8887-8895. PubMed: 11751413. 11751413

[9] Warburton C, Dragowska WH, Gelmon K, Chia S, Yan H et al. (2004) Treatment of HER-2/neu overexpressing breast cancer xenograft models with trastuzumab (Herceptin) and gefitinib (ZD1839): drug combination effects on tumor growth, HER-2/neu and epidermal growth factor receptor expression, and viable hypoxic cell fraction. Clin Cancer Res 10: 2512-2524. doi:10.1158/1078-0432.CCR-03-0244. PubMed: 15073131. 15073131

[10] Anido J, Matar P, Albanell J, Guzmán M, Rojo F et al. (2003) ZD1839, a specific epidermal growth factor receptor (EGFR) tyrosine kinase inhibitor, induces the formation of inactive EGFR/HER2 and EGFR/HER3 heterodimers and prevents heregulin signaling in HER2-overexpressing breast cancer cells. Clin Cancer Res 9: 1274-1283. PubMed: 12684395. 12684395

[11] Brehmer D, Greff Z, Godl K, Blencke S, Kurtenbach A et al. (2005) Cellular targets of gefitinib. Cancer Res 65: 379-382. PubMed: 15695376. 15695376

[12] Maiuri MC, Zalckvar E, Kimchi A, Kroemer G (2007) Self-eating and self-killing: crosstalk between autophagy and apoptosis. Nat Rev Mol Cell Biol 8: 741-752. doi:10.1038/nrm2239. PubMed: 17717517. 17717517

[13] Bampton ET, Goemans CG, Niranjan D, Mizushima N, Tolkovsky AM (2005) The dynamics of autophagy visualized in live cells: from autophagosome formation to fusion with endo/lysosomes. Autophagy 1: 23-36. PubMed: 16874023. 16874023 10.4161/auto.1.1.1495

[14] Abedin MJ, Wang D, McDonnell MA, Lehmann U, Kelekar A (2007) Autophagy delays apoptotic death in breast cancer cells following DNA damage. Cell Death Differ 14: 500-510. doi:10.1038/sj.cdd.4402039. PubMed: 16990848. 16990848

[15] Qadir MA, Kwok B, Dragowska WH, To KH, Le D et al. (2008) Macroautophagy inhibition sensitizes tamoxifen-resistant breast cancer cells and enhances mitochondrial depolarization. Breast Cancer Res Treat 112: 389-403. doi:10.1007/s10549-007-9873-4. PubMed: 18172760. 18172760

[16] Takeuchi H, Kanzawa T, Kondo Y, Kondo S (2004) Inhibition of platelet-derived growth factor signalling induces autophagy in malignant glioma cells. Br J Cancer 90: 1069-1075. doi:10.1038/sj.bjc.6601605. PubMed: 14997209. 14997209 PMC2409632

[17] Vazquez-Martin A, Oliveras-Ferraros C, Menendez JA (2009) Autophagy facilitates the development of breast cancer resistance to the anti-HER2 monoclonal antibody trastuzumab. PLOS ONE 4: e6251. doi:10.1371/journal.pone.0006251. PubMed: 19606230. 19606230 PMC2708925

[18] Han W, Pan H, Chen Y, Sun J, Wang Y et al. (2011) EGFR tyrosine kinase inhibitors activate autophagy as a cytoprotective response in human lung cancer cells. PLOS ONE 6: e18691. doi:10.1371/journal.pone.0018691. PubMed: 21655094. 21655094 PMC3107207

[19] Chen S, Li X, Feng J, Chang Y, Wang Z et al. (2011) Autophagy facilitates the Lapatinib resistance of HER2 positive breast cancer cells. Med Hypotheses 77: 206-208. doi:10.1016/j.mehy.2011.04.013. PubMed: 21570197. 21570197

[20] Harhaji-Trajkovic L, Vilimanovich U, Kravic-Stevovic T, Bumbasirevic V, Trajkovic V (2009) AMPK-mediated autophagy inhibits apoptosis in cisplatin-treated tumour cells. J Cell Mol Med 13: 3644-3654. doi:10.1111/j.1582-4934.2009.00663.x. PubMed: 20196784. 20196784 PMC4516513

[21] Li X, Lu Y, Pan T, Fan Z (2010) Roles of autophagy in cetuximab-mediated cancer therapy against EGFR. Autophagy 6: 1066-1077. doi:10.4161/auto.6.8.13366. PubMed: 20864811. 20864811 PMC3039478

[22] Sun WL, Chen J, Wang YP, Zheng H (2011) Autophagy protects breast cancer cells from epirubicin-induced apoptosis and facilitates epirubicin-resistance development. Autophagy 7: 1035-1044. doi:10.4161/auto.7.9.16521. PubMed: 21646864. 21646864

[23] Apel A, Herr I, Schwarz H, Rodemann HP, Mayer A (2008) Blocked autophagy sensitizes resistant carcinoma cells to radiation therapy. Cancer Res 68: 1485-1494. doi:10.1158/0008-5472.CAN-07-0562. PubMed: 18316613. 18316613

[24] Degtyarev M, De Mazière A, Orr C, Lin J, Lee BB et al. (2008) Akt inhibition promotes autophagy and sensitizes PTEN-null tumors to lysosomotropic agents. J Cell Biol 183: 101-116. doi:10.1083/jcb.200801099. PubMed: 18838554. 18838554 PMC2557046

[25] Gupta A, Roy S, Lazar AJ, Wang WL, McAuliffe JC et al. (2010) Autophagy inhibition and antimalarials promote cell death in gastrointestinal stromal tumor (GIST). Proc Natl Acad Sci U S A 107: 14333-14338. doi:10.1073/pnas.1000248107. PubMed: 20660757. 20660757 PMC2922542

[26] Mirzoeva OK, Hann B, Hom YK, Debnath J, Aftab D et al. (2011) Autophagy suppression promotes apoptotic cell death in response to inhibition of the PI3K-mTOR pathway in pancreatic adenocarcinoma. J Mol Med (Berl) 89: 877-889. doi:10.1007/s00109-011-0774-y. PubMed: 21678117. 21678117

[27] Ma XH, Piao S, Wang D, McAfee QW, Nathanson KL et al. (2011) Measurements of tumor cell autophagy predict invasiveness, resistance to chemotherapy, and survival in melanoma. Clin Cancer Res 17: 3478-3489. doi:10.1158/1078-0432.CCR-10-2372. PubMed: 21325076. 21325076 PMC3096713

[28] Hu YL, Jahangiri A, Delay M, Aghi MK (2012) Tumor cell autophagy as an adaptive response mediating resistance to treatments such as antiangiogenic therapy. Cancer Res 72: 4294-4299. doi:10.1158/1538-7445.AM2012-4294. PubMed: 22915758. 22915758 PMC3432684

[29] Janku F, McConkey DJ, Hong DS, Kurzrock R (2011) Autophagy as a target for anticancer therapy. Nat Rev Clin Oncol 8: 528-539. doi:10.1038/nrclinonc.2011.71. PubMed: 21587219. 21587219

[30] Maycotte P, Thorburn A (2011) Autophagy and cancer therapy. Cancer Biol Ther 11: 127-137. doi:10.4161/cbt.11.2.14627. PubMed: 21178393. 21178393 PMC3047083

[31] Amaravadi RK, Lippincott-Schwartz J, Yin XM, Weiss WA, Takebe N et al. (2011) Principles and current strategies for targeting autophagy for cancer treatment. Clin Cancer Res 17: 654-666. doi:10.1158/1078-0432.CCR-10-2634. PubMed: 21325294. 21325294 PMC3075808

[32] Swampillai AL, Salomoni P, Short SC (2012) The role of autophagy in clinical practice. Clin Oncol R Coll Radiol 24: 387-395. doi:10.1016/j.clon.2011.09.010. PubMed: 22032864. 22032864

[33] Tanner M, Kapanen AI, Junttila T, Raheem O, Grenman S et al. (2004) Characterization of a novel cell line established from a patient with Herceptin-resistant breast cancer. Mol Cancer Ther 3: 1585-1592. PubMed: 15634652. 15634652

[34] Balgi AD, Fonseca BD, Donohue E, Tsang TC, Lajoie P et al. (2009) Screen for chemical modulators of autophagy reveals novel therapeutic inhibitors of mTORC1 signaling. PLOS ONE 4: e7124. doi:10.1371/journal.pone.0007124. PubMed: 19771169. 19771169 PMC2742736

[35] Klionsky DJ, Abdalla FC, Abeliovich H, Abraham RT, Acevedo-Arozena A et al. (2012) Guidelines for the use and interpretation of assays for monitoring autophagy. Autophagy 8: 445-544. doi:10.4161/auto.19496. PubMed: 22966490. 22966490 PMC3404883

[36] Dragowska WH, Weppler SA, Qadir MA, Wong LY, Franssen Y et al. (2011) The combination of gefitinib and RAD001 inhibits growth of HER2 overexpressing breast cancer cells and tumors irrespective of trastuzumab sensitivity. BMC Cancer 11: 420. doi:10.1186/1471-2407-11-420. PubMed: 21961653. 21961653 PMC3207940

[37] Weigelt B, Warne PH, Downward J (2011) PIK3CA mutation, but not PTEN loss of function, determines the sensitivity of breast cancer cells to mTOR inhibitory drugs. Oncogene. 10.1038/onc.2011.42 21358673

[38] Köninki K, Barok M, Tanner M, Staff S, Pitkänen J et al. (2010) Multiple molecular mechanisms underlying trastuzumab and lapatinib resistance in JIMT-1 breast cancer cells. Cancer Lett 294: 211-219. doi:10.1016/j.canlet.2010.02.002. PubMed: 20193978. 20193978

[39] McKillop D, Partridge EA, Kemp JV, Spence MP, Kendrew J et al. (2005) Tumor penetration of gefitinib (Iressa), an epidermal growth factor receptor tyrosine kinase inhibitor. Mol Cancer Ther 4: 641-649. doi:10.1158/1535-7163.MCT-04-0329. PubMed: 15827338. 15827338

[40] Yoshimori T, Yamamoto A, Moriyama Y, Futai M, Tashiro Y (1991) Bafilomycin A1, a specific inhibitor of vacuolar-type H(+)-ATPase, inhibits acidification and protein degradation in lysosomes of cultured cells. J Biol Chem 266: 17707-17712. PubMed: 1832676. 1832676

[41] Wirawan E, Lippens S, Vanden Berghe T, Romagnoli A, Fimia GM et al. (2012) Beclin1: a role in membrane dynamics and beyond. Autophagy 8: 6-17. doi:10.4161/auto.8.1.16645. PubMed: 22170155. 22170155

[42] Kaiser SE, Mao K, Taherbhoy AM, Yu S, Olszewski JL et al. (2012) Noncanonical E2 recruitment by the autophagy E1 revealed by Atg7-Atg3 and Atg7-Atg10 structures. Nat Struct Mol Biol 19: 1242-1249. doi:10.1038/nsmb.2415. PubMed: 23142976. 23142976 PMC3515690

[43] Cooper RG, Magwere T (2008) Chloroquine: novel uses & manifestations. Indian J Med Res 127: 305-316. PubMed: 18577785. 18577785

[44] Swaisland HC, Smith RP, Laight A, Kerr DJ, Ranson M et al. (2005) Single-dose clinical pharmacokinetic studies of gefitinib. Clin Pharmacokinet 44: 1165-1177. doi:10.2165/00003088-200544110-00004. PubMed: 16231967. 16231967

[45] Jahanzeb M (2008) Adjuvant trastuzumab therapy for HER2-positive breast cancer. Clin Breast Cancer 8: 324-333. doi:10.3816/CBC.2008.n.037. PubMed: 18757259. 18757259

[46] Tevaarwerk AJ, Kolesar JM (2009) Lapatinib: a small-molecule inhibitor of epidermal growth factor receptor and human epidermal growth factor receptor-2 tyrosine kinases used in the treatment of breast cancer. Clin Ther 31 2: 2332-2348. PubMed: 20110044. 20110044 10.1016/j.clinthera.2009.11.029

[47] Gelmon KA, Mackey J, Verma S, Gertler SZ, Bangemann N et al. (2004) Use of trastuzumab beyond disease progression: observations from a retrospective review of case histories. Clin Breast Cancer 5: 52-62; discussion: 15140285. 15140285 10.3816/cbc.2004.n.010

[48] Nahta R, Esteva FJ (2007) Trastuzumab: triumphs and tribulations. Oncogene 26: 3637-3643. doi:10.1038/sj.onc.1210379. PubMed: 17530017. 17530017

[49] Arteaga CL, Sliwkowski MX, Osborne CK, Perez EA, Puglisi F et al. (2012) Treatment of HER2-positive breast cancer: current status and future perspectives. Nat Rev Clin Oncol 9: 16-32. PubMed: 22124364. 10.1038/nrclinonc.2011.177 22124364

[50] Rexer BN, Arteaga CL (2012) Intrinsic and acquired resistance to HER2-targeted therapies in HER2 gene-amplified breast cancer: mechanisms and clinical implications. Crit Rev Oncog 17: 1-16. doi:10.1615/CritRevOncog.v17.i1.20. PubMed: 22471661. 22471661 PMC3394454

[51] Bernardini M, Lee CH, Beheshti B, Prasad M, Albert M et al. (2005) High-resolution mapping of genomic imbalance and identification of gene expression profiles associated with differential chemotherapy response in serous epithelial ovarian cancer. Neoplasia 7: 603-613. doi:10.1593/neo.04760. PubMed: 16036111. 16036111 PMC1501280

[52] Prasad M, Bernardini M, Tsalenko A, Marrano P, Paderova J et al. (2008) High definition cytogenetics and oligonucleotide aCGH analyses of cisplatin-resistant ovarian cancer cells. Genes Chromosomes Cancer 47: 427-436. doi:10.1002/gcc.20547. PubMed: 18273836. 18273836

[53] Lipinski MM, Hoffman G, Ng A, Zhou W, Py BF et al. (2010) A genome-wide siRNA screen reveals multiple mTORC1 independent signaling pathways regulating autophagy under normal nutritional conditions. Dev Cell 18: 1041-1052. doi:10.1016/j.devcel.2010.05.005. PubMed: 20627085. 20627085 PMC2935848

[54] Zou Y, Ling YH, Sironi J, Schwartz EL, Perez-Soler R et al. (2013) The autophagy inhibitor chloroquine overcomes the innate resistance of wild-type EGFR non-small-cell lung cancer cells to erlotinib. J Thorac Oncol 8: 693-702. doi:10.1097/JTO.0b013e31828c7210. PubMed: 23575415. 23575415 PMC3855301

[55] Weihua Z, Tsan R, Huang WC, Wu Q, Chiu CH et al. (2008) Survival of cancer cells is maintained by EGFR independent of its kinase activity. Cancer Cell 13: 385-393. doi:10.1016/j.ccr.2008.03.015. PubMed: 18455122. 18455122 PMC2413063

[56] Zhang L, Gjoerup O, Roberts TM (2004) The serine/threonine kinase cyclin G-associated kinase regulates epidermal growth factor receptor signaling. Proc Natl Acad Sci U S A 101: 10296-10301. doi:10.1073/pnas.0403175101. PubMed: 15240878. 15240878 PMC478566

[57] Eisenberg-Lerner A, Bialik S, Simon HU, Kimchi A (2009) Life and death partners: apoptosis, autophagy and the cross-talk between them. Cell Death Differ 16: 966-975. doi:10.1038/cdd.2009.33. PubMed: 19325568. 19325568

[58] Kanzawa T, Germano IM, Komata T, Ito H, Kondo Y et al. (2004) Role of autophagy in temozolomide-induced cytotoxicity for malignant glioma cells. Cell Death Differ 11: 448-457. doi:10.1038/sj.cdd.4401359. PubMed: 14713959. 14713959

[59] Shingu T, Fujiwara K, Bögler O, Akiyama Y, Moritake K et al. (2009) Inhibition of autophagy at a late stage enhances imatinib-induced cytotoxicity in human malignant glioma cells. Int J Cancer 124: 1060-1071. doi:10.1002/ijc.24030. PubMed: 19048625. 19048625

[60] Maycotte P, Aryal S, Cummings CT, Thorburn J, Morgan MJ et al. (2012) Chloroquine sensitizes breast cancer cells to chemotherapy independent of autophagy. Autophagy 8: 200-212. doi:10.4161/auto.8.2.18554. PubMed: 22252008. 22252008 PMC3336076

[61] Boya P, Gonzalez-Polo RA, Poncet D, Andreau K, Vieira HL et al. (2003) Mitochondrial membrane permeabilization is a critical step of lysosome-initiated apoptosis induced by hydroxychloroquine. Oncogene 22: 3927-3936. doi:10.1038/sj.onc.1206622. PubMed: 12813466. 12813466

[62] Bristol ML, Emery SM, Maycotte P, Thorburn A, Chakradeo S et al. (2013) Autophagy inhibition for chemosensitization and radiosensitization in cancer: do the preclinical data support this therapeutic strategy? J Pharmacol Exp Ther, 344: 544–52. PubMed: 23291713. 23291713 10.1124/jpet.112.199802 PMC3583507

[63] Lamoureux F, Thomas C, Crafter C, Kumano M, Zhang F et al. (2013) Blocked autophagy using lysosomotropic agents sensitizes resistant prostate tumor cells to the novel Akt inhibitor AZD5363. Clin Cancer Res 19: 833-844. doi:10.1158/1078-0432.CCR-12-3114. PubMed: 23258740. 23258740

[64] Aita VM, Liang XH, Murty VV, Pincus DL, Yu W et al. (1999) Cloning and genomic organization of beclin 1, a candidate tumor suppressor gene on chromosome 17q21. Genomics 59: 59-65. doi:10.1006/geno.1999.5851. PubMed: 10395800. 10395800

[65] Saito H, Inazawa J, Saito S, Kasumi F, Koi S et al. (1993) Detailed deletion mapping of chromosome 17q in ovarian and breast cancers: 2-cM region on 17q21.3 often and commonly deleted in tumors. Cancer Res 53: 3382-3385. PubMed: 8100738. 8100738

