# Supplementary figures and images for: Induction of Autophagy Is an Early Response to Gefitinib and a Potential Therapeutic Target in Breast Cancer
Source: PLoS One. 2013 Oct 11;8(10):e76503. doi: 10.1371/journal.pone.0076503 (PMC3795739; doi:10.1371/journal.pone.0076503)

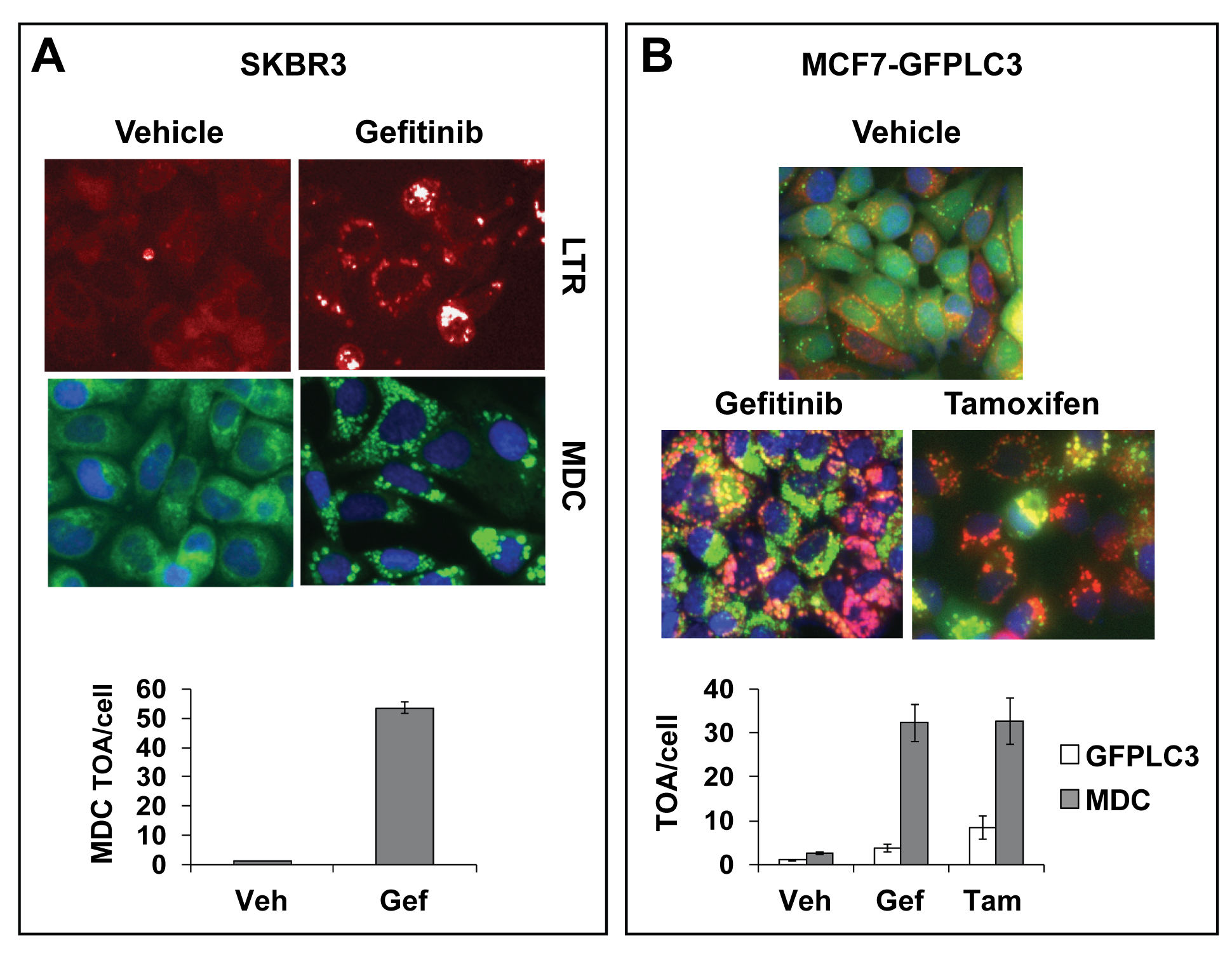

Supplement: Figure S1 — Quantitation of autophagy-associated organelles by HCA methods in SKBR3 and MCF7-GFPLC3 cells treated with vehicle, gefitinib or tamoxifen. (A) Representative images obtained with IN Cell 1000 Analyzer of SKBR3 cells treated for 48 h with vehicle or 10 μM gefitinib stained with lysotracker red (LTR) (red) or MDC (green) and counterstained with DRAQ5 (blue). Bottom graph: the average MDC TOA/cell (mean±SD, n = 6 replicate wells) obtained with HCA in vehicle (Veh) or gefitinib (Gef) treated cells. (B) Representative images of MCF7-GFPLC3 cells treated for 48 h with vehicle, 20 μM gefitinib or 10 μM tamoxifen stained with Hoechst 33342 (blue) and LTR (red). Green puncta represent GFPLC3-labeled autophagosomes, red puncta represent lysosomes and yellow puncta represent autolysosomes. Bottom graph: the average MDC and GFPLC3 TOA/cell (mean±SD, n = 6 replicate wells) obtained with HCA in vehicle (Veh), gefitinib (Gef) or tamoxifen (Tam) treated cells. (TIF) [file pone.0076503.s001.tif]

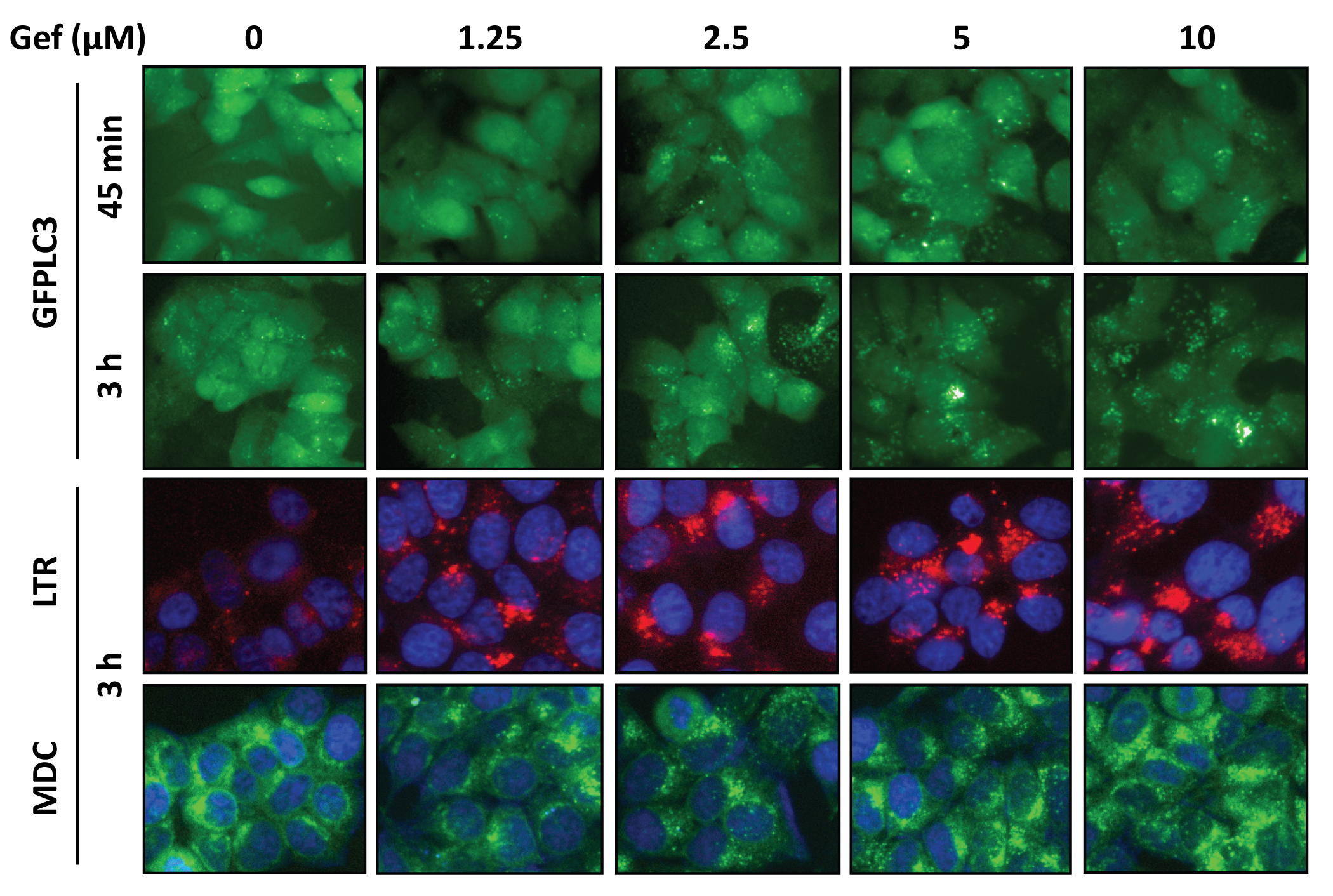

Supplement: Figure S2 — The dynamics of autophagy-associated organelle formation in MCF7-GFPLC3 cells treated with gefitinib. Representative images of MCF7-GFPLC3 cells treated with vehicle (0 µM gefitinib) or indicated gefitinib concentrations acquired with IN Cell 1000. GFPLC3 panel: the green background in the control cells represents the GFPLC3 protein which is diffusely spread throughout the cytoplasm. With time the GFPLC3 staining becomes more defined and GFPLC3-labeled organelles (green puncta) marking the location of autophagosome membrane associated LC3-II protein are observed in cells. LTR panel: images of MCF7-GFPLC3 cells stained with Hoechst 33342 (blue nuclei) and lysotracker red (LTR; red puncta). MDC panel: images of MCF7-GFPLC3 cells stained with DRAQ5 (blue) and MDC (green puncta) in the cellular cytoplasm. Images were pseudo-colored and overlaid using the Investigator software. (TIF) [file pone.0076503.s002.tif]

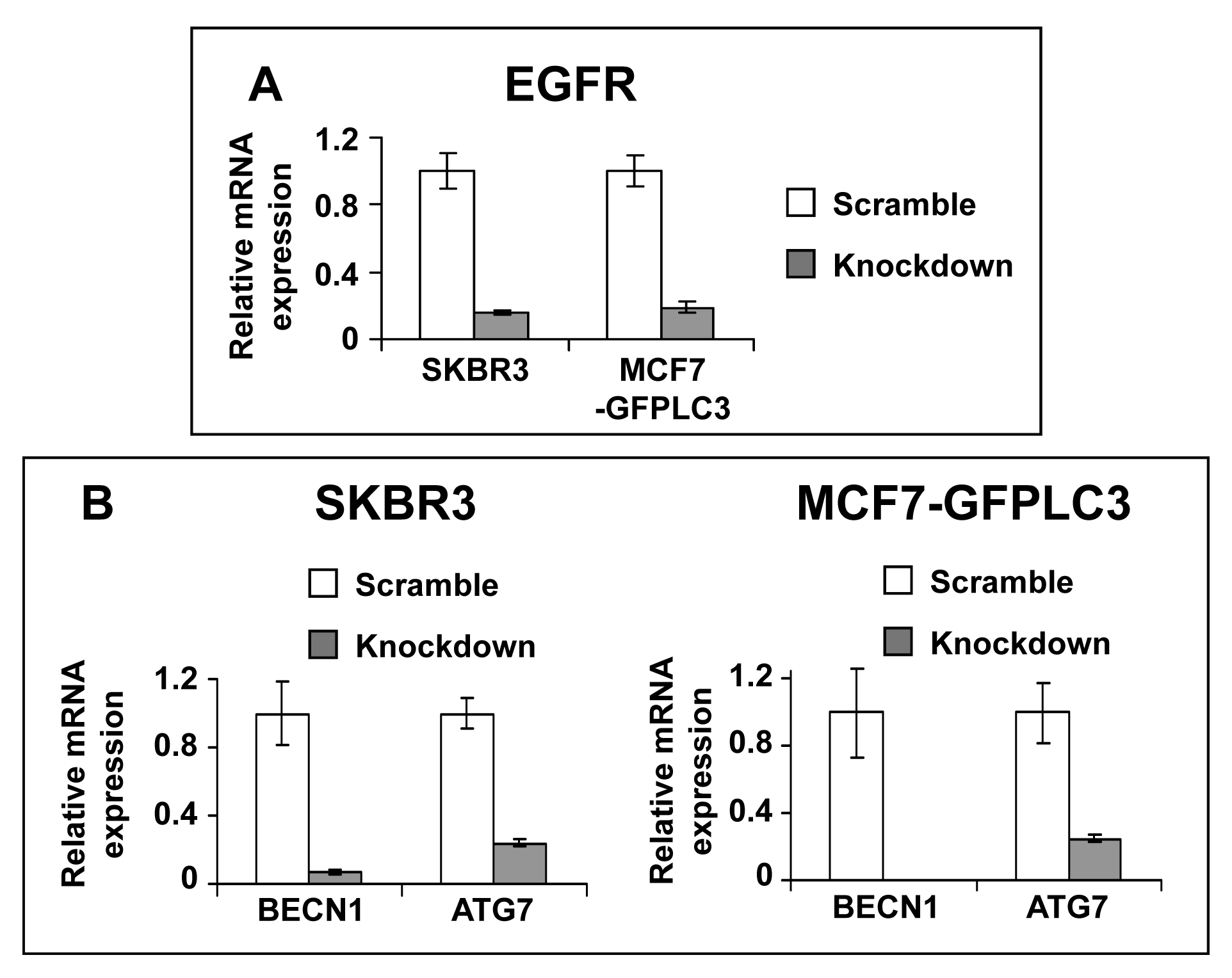

Supplement: Figure S3 — Validation of siRNA-mediated knockdown by qRT-PCR. (A) Levels of EGFR mRNA in SKBR3 cells harvested 72 h post knockdown and in MCF7-GFPLC3 cells harvested 48 h post double knockdown. (B) Levels of BECN1 and ATG7 mRNA in SKBR3 and MCF7-GFPLC3 cells harvested 72 h post knockdown. mRNA expression for each of the indicated genes in (A) and (B) is shown relative to the scrambled non-silencing siRNA control expressed as 1. Each data point represents a mean±SD from 3 replicate PCR samples. (TIF) [file pone.0076503.s003.tif]
